# Integrating Molecular Dynamics and Machine Learning for Solvation‐Guided Electrolyte Optimization in Lithium Metal Batteries

**DOI:** 10.1002/advs.202504997

**Published:** 2025-06-30

**Authors:** Xiwang Chang, Yang Yang, Weiheng Xu, Zhe Wang, Wenhan Li, Hongda Gao, Dubin Huang, Aijun Li, Yaofeng Zhu

**Affiliations:** ^1^ School of Materials Science & Engineering Zhejiang Sci‐Tech University Hangzhou 310018 China; ^2^ School of Materials Science and Engineering, and Academy for Advanced Interdisciplinary Studies Peking University Beijing 100871 China; ^3^ Zhejiang Golden Feather New Energy Technology Co., Ltd Huzhou 313300 China

**Keywords:** electrolyte, lithium metal battery, machine learning, molecular dynamics, optimal trend

## Abstract

Optimizing liquid electrolytes is essential for achieving long‐term cycling stability and high safety in lithium metal batteries. However, severe side reactions and lithium dendrite formation during repeated cycling lead to low Coulombic efficiency (CE) and limited lifespan. Herein, a rapid and cost‐effective strategy that integrates high‐throughput molecular dynamics simulations with machine learning predictions is proposed, further validated through experimental evaluation. A mixed electrolyte composed of LiFSI (LiN(SO_2_F)_2_) as the main salt, DEE (1,2‐diethoxyethane) as the solvent, and LiNFS (LiC_4_F_9_SO_3_) as an additive achieves a significantly improved CE of 98.32%. Key molecular descriptors are identified for each performance label, and the most accurate model is selected through rigorous benchmarking. The optimal region reveals a preference for medium‐to‐high salt concentrations; low C, O, and N content; and high F content in salts, along with high C and low O content in solvents. This framework enables reusable and resource‐efficient modeling for targeted electrolyte design and accelerated optimization.

## Graphical abstract

A data‐driven workflow integrating molecular dynamics simulations and machine learning enables efficient discovery of optimized lithium battery electrolytes. Key structural descriptors are screened for predicting coordination behavior and ion transport. Gaussian process regression identifies an optimal feature space, guiding rational electrolyte design. Experimental validation confirms enhanced electrochemical performance in both half‐cell and full‐cell configurations.

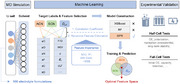

## 1 Introduction

The rapid evolution of contemporary energy storage technologies has been largely driven by lithium‐ion batteries (LIBs), which deliver energy densities approaching 300 Wh kg^−1^ and have become the cornerstone for portable electronics and electric vehicles.^[^ ^1^ ^]^ However, the limited theoretical capacity of graphite anodes (372 mAh g^−1^) increasingly constrains the performance ceiling of LIBs. This challenge has stimulated extensive research interest in lithium‐metal batteries (LMBs), which utilize lithium metal as the anode—a material that offers an ultrahigh theoretical capacity of 3860 mAh g^−1^ and the lowest redox potential (−3.04 V vs SHE), making it a highly promising candidate for next‐generation energy storage.^[^ ^2^ ^]^ When paired with advanced cathodes such as LiNi*_x_* Co*_y_* Mn*_z_* O_2_ (NCM) or LiFePO_4_, LMBs can deliver outstanding energy metrics, with gravimetric and volumetric energy densities reaching ≈450 Wh kg^−1^ and ≈1200 Wh L^−1^, respectively.^[^ ^3^ ^]^

Despite these advantages, the highly reactive nature of lithium metal poses significant interfacial challenges. Its strong reductive potential leads to parasitic reactions with conventional carbonate‐based electrolytes, resulting in low CE, unstable solid—electrolyte interphase (SEI) formation, dendritic lithium growth, and rapid capacity degradation.^[^ ^4^ ^]^ To address these issues, ether‐based electrolytes featuring ─CH_2_CH_2_O─ backbones have emerged as promising alternatives due to their superior reductive stability and high ionic conductivity.^[^ ^5^ ^]^ Nonetheless, single‐component ether solvents (e.g., DME, diglyme) still suffer from several intrinsic limitations, including decomposition into lithium alkoxides (ROLi), unstable SEI formation, and gradual CE deterioration during cycling.^[^ ^6^ ^]^

To unlock the practical viability of LMBs, rational electrolyte design has become a key enabler. Recent advances in electrolyte engineering extend beyond conventional formulations and now encompass high‐concentration and localized high‐concentration electrolytes (LHCEs),^[^ ^7^ ^]^ entropy‐stabilized multi‐solvent systems,^[^ ^8^ ^]^ fluorinated weakly solvating solvents,^[^ ^9^ ^]^ and functional additives for interfacial modulation.^[^ ^10^ ^]^ However, traditional trial‐and‐error strategies remain inefficient, costly, and often unable to keep pace with the expanding compositional complexity required for next‐generation LMBs.

To accelerate discovery, data‐driven approaches combining molecular dynamics (MD) simulations with machine learning (ML) have recently gained traction. MD simulations enable atomistic insights into solvation structures and ion transport behaviors; while, ML can extract patterns and predict electrolyte performance based on physicochemical descriptors. For example, Cui et al.^[^ ^11^ ^]^ employed ML to uncover correlations between electrolyte composition and CE, leading to the identification of four promising formulations. Gao et al.^[^ ^12^ ^]^ integrated ML with density functional theory (DFT) to predict donor numbers of polymer segments and used MD to validate the effects on lithium‐ion transport. Yuan et al.^[^ ^13^ ^]^ applied quantum calculations and interpretable ML to reveal structure—property relationships in fluorinated ethers, establishing design principles for next‐generation fluoroether electrolytes.

**Figure 1:**
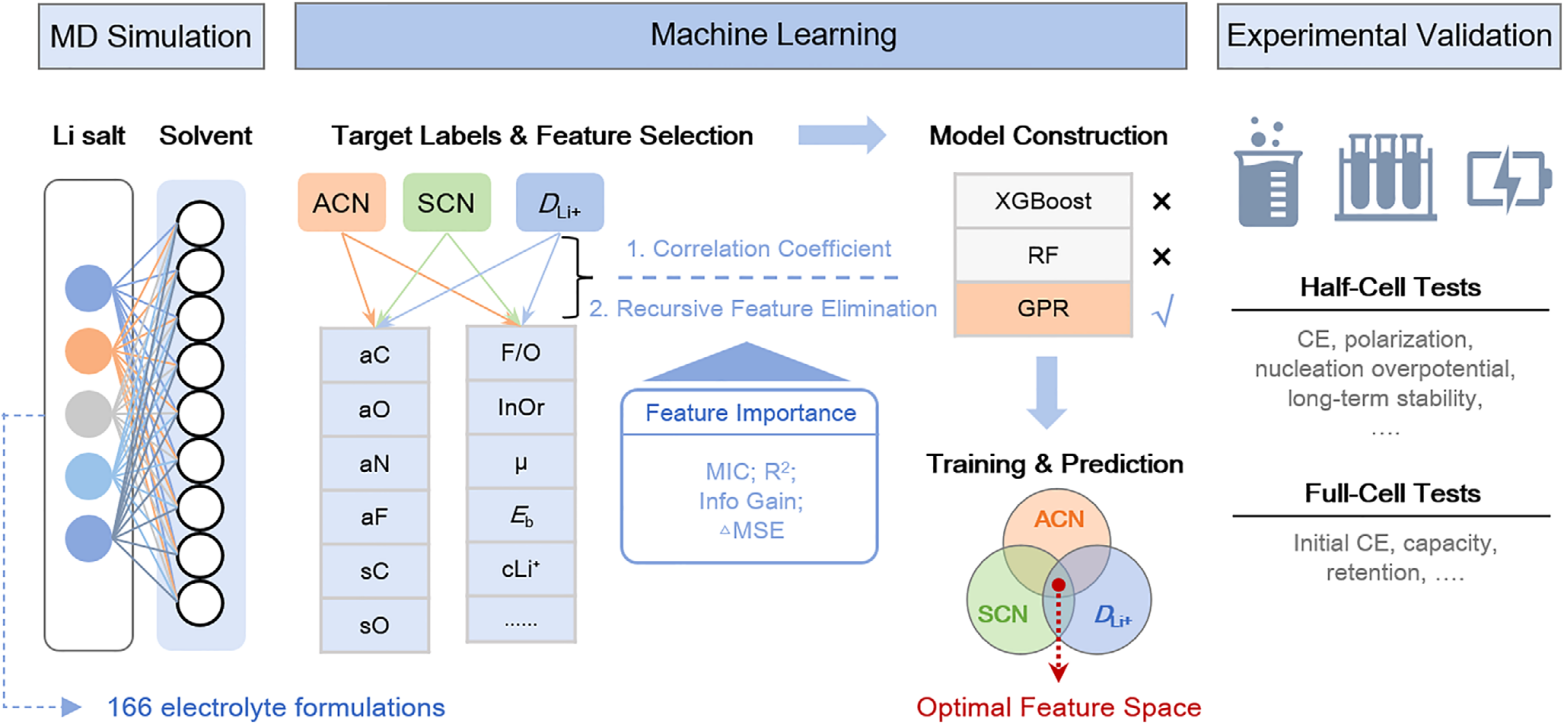
Integrated workflow combining MD simulation, machine learning, and experimental validation for electrolyte optimization. The process begins with solvation structure analysis across 166 Li salt–solvent systems using MD simulations. Key features are extracted and selected for three performance targets: anion coordination number (ACN), solvent coordination number (SCN), and *D*_Li_+. After model construction and benchmarking, Gaussian Process Regression (GPR) is identified as the optimal algorithm. High‐performing predictions are mapped to an optimal feature space, guiding the rational design of electrolytes. Selected formulations are subsequently validated in half‐cell and full‐cell configurations.

In this study, we propose a synergistic computational–experimental framework to accelerate electrolyte discovery for high‐performance LMBs (**Figure** **1**). First, MD simulations were conducted to investigate solvation structures and lithium‐ion diffusion coefficients (*D*_Li⁺_) for 166 unique combinations of five lithium salts and eight ether‐based solvents (Figure S1 and Table S1, Supporting Information).^[^ ^7^, ^10^, ^14^ ^]^ This dataset was used to construct an ML training set. To overcome overfitting challenges posed by the limited dataset size, robust feature selection and model optimization were carried out. Among several algorithms evaluated, Gaussian process regression (GPR) exhibited the best predictive performance. The trained GPR model was then used to screen for optimal composition trends. Guided by computational predictions, eleven candidate electrolytes were synthesized and evaluated. Among them, a ternary system comprising LiFSI (lithium bis(fluorosulfonyl)imide) as the primary salt, DEE (1,2‐diethoxyethane) as the primary solvent, and LiNFS (lithium nonafluorobutanesulfonate) as an additive exhibited the highest Coulombic efficiency (98.32%) and the lowest overpotential, demonstrating the power of integrated design methodologies for advanced electrolyte development.

## 2 Results and Discussion

### 2.1 MD Simulation Results and Sample Set Construction

**Figure 2:**
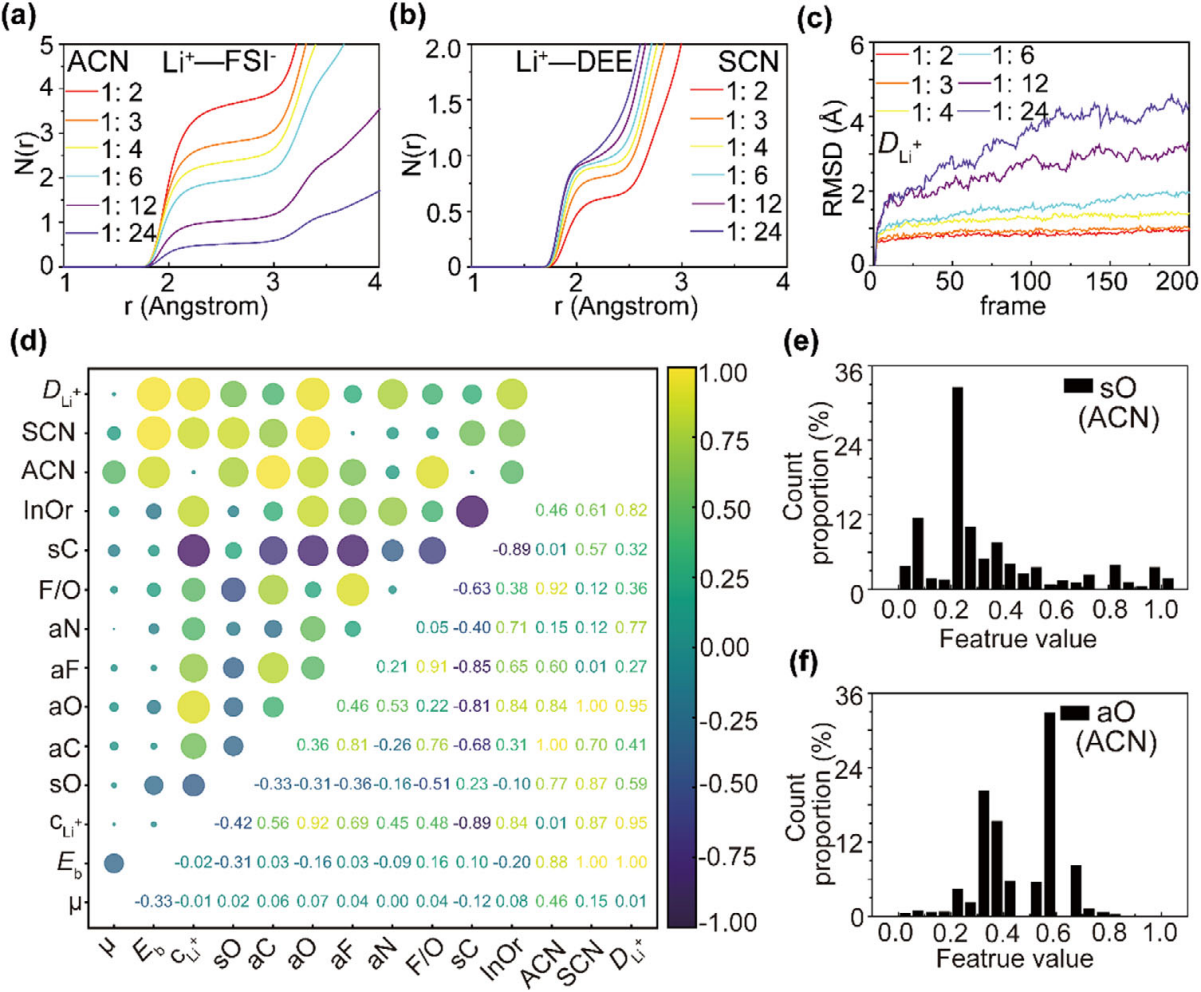
Coordination chemistry and feature optimization in electrolyte systems. a–c) Evolution of coordination properties and transport behavior in LiFSI–DEE electrolytes: anion coordination number (ACN, Li⁺–FSI−) (a), solvent coordination number (SCN, Li⁺–DEE) (b), and root mean square displacement (RMSD) of Li⁺ at various concentrations (Li⁺:O(solvent) = 1:2 to 1:24), showing opposing trends between anion/solvent coordination and ion mobility (c). d) Feature correlation matrix (left) and feature importance heatmap (right) across 11 molecular descriptors and 3 target labels (ACN, SCN, and *D*_Li_+); feature importance was reduced to [0.01, 1] using Equation (S1), Supporting Information. e,f) Frequency histograms of top‐ranked features within the ACN‐optimized sample space, highlighting preferred ranges for: solvent oxygen content (sO) (e) and anion oxygen content (aO) (f); for predicted feature value frequency count proportion of sO and aO in ACN set, the suggested section was sO: [0.2, 0.55] aO: [0.25, 0.4], respectively. Abbreviations: µ: dipole moment of solvents; aC, aO, aN, aF: molar fraction of C, O, N, and F in anions; sC, sO: molar fraction of C and O in solvents; F/O: fluorine‐to‐oxygen molar ratio; and InOr: inorganic‐to‐organic molar ratio (excluding H and Li).

As illustrated in **Figure** **2**, panels (a), (b), and (c) present MD simulation results for the LiFSI–DEE electrolyte system. In panel (a), the ACN—corresponding to the coordination between Li^+^ and FSI^−^—decreases systematically with dilution as the Li^+^:O(solvent) molar ratio increases from 1: 2 to 1: 24. Conversely, panel (b) shows the SCN for Li^+^–DEE interactions, which increases with dilution. Similarly, panel (c) displays the root mean square displacement (RMSD) of Li^+^ ions, a proxy for diffusion behavior, which also increases as concentration decreases. These trends—namely, increasing SCN and RMSD but decreasing ACN with dilution—are consistently observed across all electrolyte systems studied (see Figures S2–S27, Supporting Information). This suggests that, regardless of the salt or solvent species, solvation structure evolution follows a universal trend governed by the relative availability of anions and solvent molecules.

To validate the reliability of our MD‐derived coordination trends, experimental characterization was conducted. As shown in Figures S28–S31, Supporting Information, the computed electrolyte densities using the CHARMM force field exhibited strong agreement with measured values, indicating accurate reproduction of macroscopic electrolyte properties. Moreover, Raman and FT‐IR spectroscopy revealed concentration‐dependent shifts in solvation structure, which closely mirrored MD‐derived coordination number trends. These results confirm that our simulations accurately captured the dynamic solvation behavior of Li^+^ across diverse concentration regimes.

In addition to coordination analysis, we evaluated the first solvation sheath radius of Li^+^ to isolate the ACN and SCN values within the primary coordination shell, in line with previous studies.^[^ ^15^ ^]^ These local coordination numbers were extracted as structural descriptors for downstream ML modeling. Further, *D*_Li_+ was computed as outlined in Section 4.1. Given the low absolute magnitudes of *D*_Li_+, the amplification for *D*_Li_+ was applied using Equation (2) to improve ML model training stability.

Based on the above analysis, three structured sample sets were constructed, each comprising 166 samples. The target labels for ML were: i) ACN within the first solvation shell, ii) SCN within the first solvation shell, and iii) log‐transformed Li^+^ diffusivity (*V*_diff_). These datasets served as the foundation for feature selection, model development, and predictive screening discussed in subsequent sections.

### 2.2 Machine Learning Prediction

#### 2.2.1 Feature Selection

##### Anion Coordination Number (ACN)

Enhancing the ACN is essential for promoting thin, inorganic‐rich, and stable SEI layers.^[^ ^16^ ^]^ To identify the most relevant input descriptors for ACN prediction, a dual strategy combining Pearson correlation and recursive feature elimination (RFE) was employed. Initial Pearson analysis revealed eight linearly correlated features with ACN: µ, *E*_b_, c_Li⁺_, sO, aO, aF, aN, and InOr. RFE was subsequently conducted using four distinct importance metrics: RF‐MIC: maximized information coefficient (non‐linear dependence); XGBoost‐IG: feature ranking via information gain; GPR‐ΔMSE: change in model error upon feature removal; and RF‐R^2^: model performance stability across folds. This multi‐metric approach revealed that *E*_b_, sO, and aO consistently ranked highly; while, additional features such as aC and F/O were elevated by non‐linear selection metrics. Based on cumulative ranking scores (normalized > 0.5, i.e., rank sum >16.5; see Figure 2d; Table S2, Supporting Information), six features were selected as optimal predictors for ACN: *E*_b_, sO, aC, aO, aF, and F/O.

##### Solvent Coordination Number (SCN)

Feature selection for SCN follows the same consensus‐ranking protocol (Figure 2d, SCN column; Table S3, Supporting Information), yielding seven descriptors with strong relevance: *E*_b_, c_Li_+, sO, aC, aO, sC, and InOr. SCN reflects solvent spatial occupancy in the primary Li^+^ solvation shell and directly influences organic SEI formation and interfacial reactivity.^[^ ^17^ ^]^ Among the descriptors, sO characterizes the number of accessible coordination sites per solvent molecule; while, *E*_b_ governs Li^+^–solvent binding strength. Anionic descriptors such as aO, aC, and c_Li_+ are also retained. Specifically, aO relates to anion oxygen content and modulates Li^+^–anion coordination; while, aC reflects anion structure, influencing competitive solvation behavior.^[^ ^18^ ^]^ Prior studies have shown SCN to be highly sensitive to solvent molecular architecture, where sC accounts for carbon content and captures the effect of chemical substitutions (e.g., fluorination,^[^ ^14^, ^19^ ^]^ methylation,^[^ ^20^ ^]^ steric hindrance,^[^ ^14^, ^21^ ^]^ and chain segment variations such as ─(CH_2_CH_2_O)─ units^[^ ^14l,m^ ^]^). InOr, a composite metric reflecting inorganic/organic composition ratios, effectively encapsulates the structural diversity arising from these modifications. Collectively, the selected features demonstrate strong physical interpretability and predictive relevance for SCN modeling.

##### Li⁺ Diffusion Coefficient (D_Li_+)

Feature selection for *D*_Li_+ is performed as detailed in Table S4, Supporting Information, identifying six optimal descriptors with ranking scores exceeding the 0.5 threshold in Figure 2d (*D*_Li_+ column): *E*_b_, c_Li_+, sO, aO, aN, and InOr. In dilute electrolytes, Li^+^ transport is dominated by solvent‐coordinated migration, with *D*_Li_+ largely governed by solvation strength and sheath structure.^[^ ^14n^ ^]^ As the concentration increases, Li^+^ diffusion becomes increasingly constrained by ion aggregation and the formation of CIPs or aggregates (AGGs), ultimately resulting in solvent entrapment within anion‐rich domains—a hallmark of “water‐in‐salt” electrolyte behavior.^[^ ^4^, ^22^ ^]^ The features InOr and c_Li_+ jointly reflect this transition from solvent‐dominated to anion‐network‐mediated transport. In particular, InOr captures the shift in solvation structure complexity and mobility constraint.^[^ ^11^ ^]^ In addition, the descriptor aN, which distinguishes between imide‐type (e.g., FSI^−^ and TFSI^−^) and non‐imide salts, provides insight into how anion functionality impacts Li^+^ mobility and electrostatic interactions. This feature set robustly captures the compositional and structural factors affecting Li^+^ transport kinetics, confirming its suitability for predictive modeling of *D*_Li_+.

#### 2.2.2 Model Development and Evaluation

To identify the most suitable machine learning model for predicting the ACN, SCN, and *D*_Li_+, a comprehensive model evaluation framework was established. Model performance was assessed based on three criteria: i) coefficient of determination (*R* ^2^) for both training and validation sets, ii) standard deviation of prediction errors, and iii) correlation coefficients between predicted and true values on the test set. All evaluations were conducted using a tenfold cross‐validation scheme to ensure statistical robustness.

**Figure 3:**
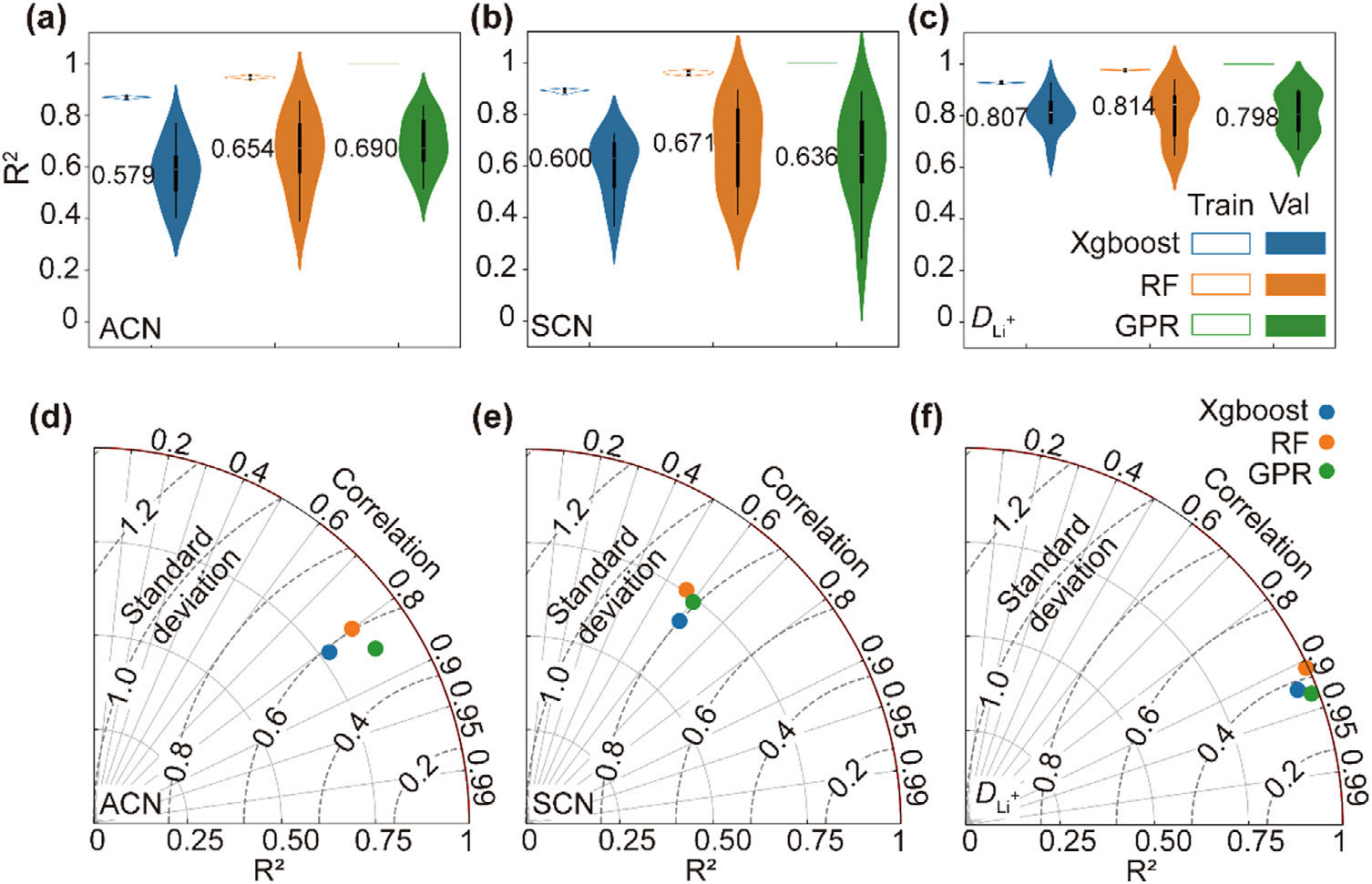
Comparative performance evaluation of machine learning models for predicting ACN, SCN, and *D*_Li_+. a–c) Distribution of *R* ^2^ values for XGBoost, RF, and GPR across training and validation sets for ACN, SCN, and *D*_Li_+, respectively. Median *R* ^2^ values for each model are annotated. d–f) Taylor plots showing performance metrics on the test sets: coefficient of determination (*R* ^2^), standard deviation, and Pearson correlation coefficient. The red dashed line indicates the ideal *R* ^2^ value of 1. GPR demonstrates the best overall performance, exhibiting the highest predictive accuracy and generalization capability across all three target properties.

**Figure** **3**a–c presents the distributions of *R* ^2^ values for three machine learning algorithms—XGBoost, RF, and GPR—on the training and validation sets for each of the three targets (ACN, SCN, and *D*_Li_+). The numerical values indicate the median *R* ^2^ on the validation set. All models achieved high *R* ^2^ values on the training sets; however, GPR consistently produced validation *R* ^2^ distributions closest to unity with minimal overfitting. For SCN, XGBoost slightly outperformed GPR in median *R* ^2^, but GPR demonstrated better generalization overall.

Figure 3d–f further quantifies model performance on the test sets, displaying *R* ^2^ values, prediction standard deviations, and correlation coefficients. The red dashed line denotes the ideal *R* ^2^ value of 1.0. For ACN prediction, GPR showed superior accuracy, achieving the highest *R* ^2^, strongest correlation, and lowest standard deviation. For SCN, although RF achieved the highest *R* ^2^, its correlation and error spread were less favorable than those of GPR. For *D*_Li_+, GPR again outperformed other models in terms of consistency and predictive reliability.

Based on this holistic comparison, GPR was selected as the optimal model for downstream prediction and trend analysis across all three electrolyte property targets.

#### 2.2.3 Prediction and Intersection Analysis

Using the methodology described in Section 4.2.4, individual predictions were performed for ACN, SCN, and *D*_Li_+ using the trained GPR models. The predicted dataset for ACN included 2.26 × 10⁷ samples, with values ranging from 0.52 to 3.85. Notably, a significant proportion of samples exhibited low ACN values. For SCN, the dataset comprised 2.24 × 10⁸ samples spanning a range from 0.19 to 1.81. The *D*_Li_+ dataset contained 2.06 × 10⁷ samples, with predicted values (*V*_diff_) ranging from 5.41 to 8.61, again mirroring the problem observed in the ACN dataset. From a physicochemical perspective, optimized solvation structures for lithium‐ion transport require: ACN > 1.5 to promote anion‐rich environments and enable AGG formation,^[^ ^23^ ^]^ SCN < 1.0 to ensure sufficient steric accessibility for anion coordination,^[^ ^14^, ^23^ ^]^ and *V*_diff_ < 7.5, which corresponds to the median *D*_Li_+ value and serves as a threshold for acceptable ion mobility. Applying these thresholds, the sample sizes were filtered down to 1.44 × 10⁷, 8.26 × 10⁷, and 1.02 × 10⁷ for ACN, SCN, and *D*_Li_+, respectively.

**Figure 4:**
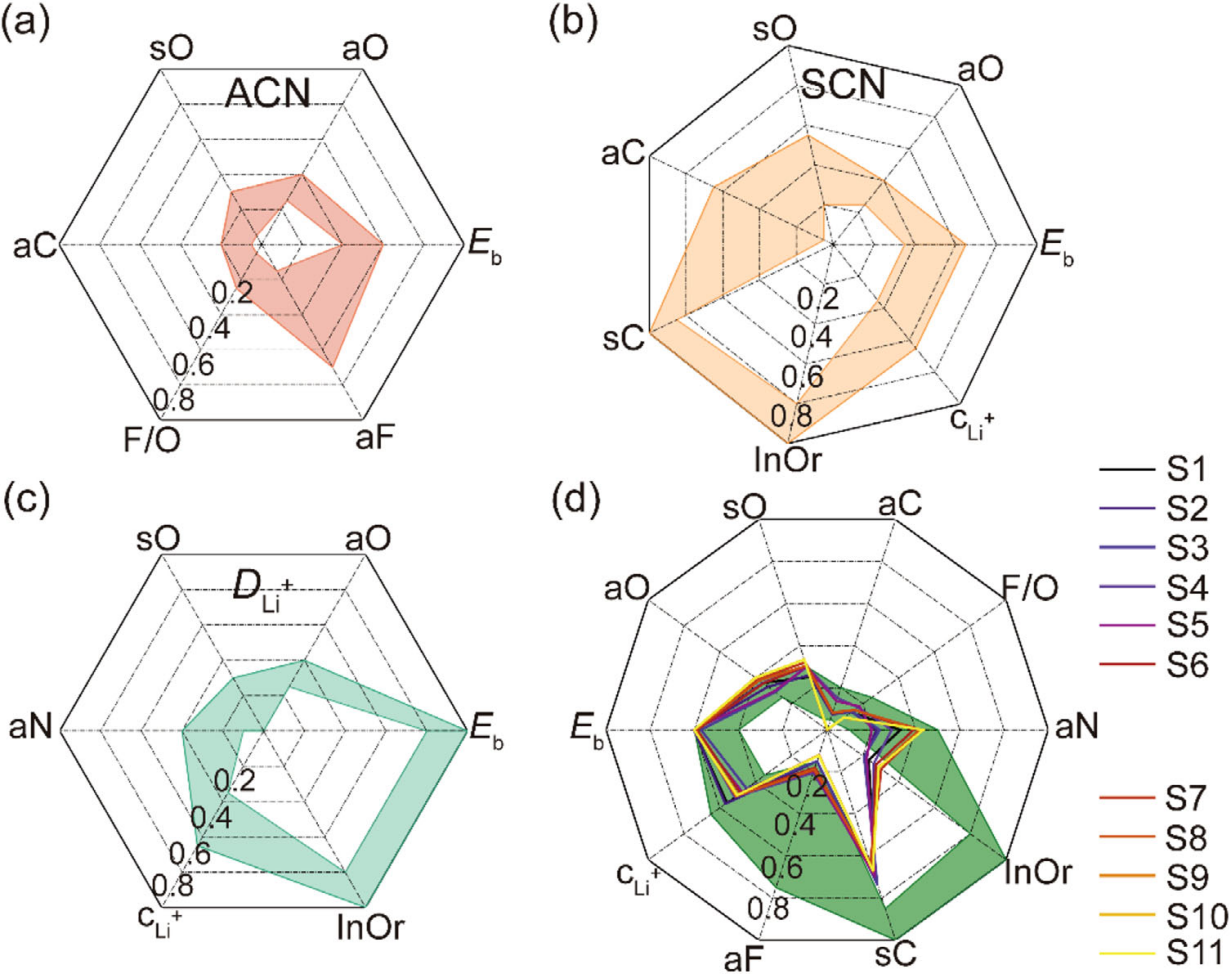
Identification of the ensemble optimal feature space for electrolyte design across three target properties. a–c) Radar plots showing the optimal feature ranges for ACN, SCN, and *D*_Li_+, respectively, derived from predictive modeling and frequency‐based feature analysis. d) Consolidated ensemble optimal feature space constructed by integrating overlapping feature ranges across all three targets. Colored lines represent the eleven experimentally formulated electrolytes (Table S5, Supporting Information) designed based on this optimal parameter space.

To identify promising electrolyte formulations that simultaneously satisfy all three target properties, the intersections among the three filtered datasets were computed based on shared values for key features: *E*_b_, sO, and aO. This intersection yielded 33 009, 11 558, and 8917 shared samples, respectively. For each intersected dataset, the frequency distribution of all feature values was statistically analyzed (see Figure 2e,f; Figures S32–S34, Supporting Information). The most frequently occurring values (modal bins) were designated as the recommended operating ranges for each feature. Using these results, three optimal feature domains were derived for ACN, SCN, and *D*_Li_+, respectively, and are illustrated in **Figure** **4**a–c.

Finally, these three optimal domains were integrated to construct a unified ensemble optimal feature space, encompassing ten features (Figure 4d). For features common to all three optimal domains (i.e., *E*_b_, sO, aO, aC, InOr, and c_Li_+), only the overlapping regions of their recommended ranges were retained. For the remaining features (F/O, aF, sC, and aN), the union of their recommended ranges across the individual models was adopted. This ensemble feature space served as the design reference for experimental electrolyte formulations.

### 2.3 Trend Analysis

As shown in Figure 4d; Table S6, Supporting Information, the recommended range for the *E*_b_ feature value lies between 0.4 and 0.6, corresponding to an actual range of −101.66 to −80.84 kcal mol^−^^1^. Within this range, a lower absolute *E*_b_ reflects weaker Li^+^–solvent interactions, whereas a higher absolute *E*_b_ indicates stronger binding. In this study, for mixed‐solvent systems, the effective *E*_b_ was estimated by a mole‐fraction‐weighted average of the component solvents’ individual *E*_b_ values. This intermediate *E*_b_ range is desirable: it ensures sufficient solvation of lithium salts; while, avoiding excessive stabilization that could lead to strongly bound ion pairs or hinder the desolvation process essential for efficient Li^+^ transport.^[^ ^14^, ^16^, ^20^ ^]^

Solvent‐related descriptors, sO and sC, were used to evaluate structural features influencing Li^+^ solvation. The optimal range for sC centers around the median of the original dataset, whereas sO favors lower values, suggesting that solvents with reduced oxygen content are preferred. This trend supports the use of structural modifications such as increased methyl substitution, ethyl terminal groups, and oxygen‐reducing strategies to attenuate solvation strength.^[^ ^14^, ^20^, ^24^ ^]^ These adjustments may indirectly increase the ACN by reducing solvent competitiveness with anions for Li^+^ coordination. For example, while DPE meets some of these criteria, its sC exceeds the recommended range and its *E*_b_ falls below the minimum threshold. Thus, solvents with moderate‐to‐high oxygen content but lower carbon content may be more favorable for further tuning.

Features prefixed with “a” (i.e., aC, aO, aN, and aF) represent compositional characteristics of the lithium salt. Their recommended ranges—[0.05, 0.20] for aC, [0.25, 0.40] for aO, [0.10, 0.50] for aN, and [0.15, 0.75] for aF—suggest that optimal salts should exhibit low carbon, moderate oxygen and nitrogen, and relatively high fluorine content. In particular, the presence of oxygen within anions promotes coordination with Li^+^; while, higher O content may enhance AGG and CIP formation.^[^ ^25^ ^]^ For fluoroalkyl‐based anions, increasing the fluorine and carbon content (e.g., more ─CF_2_─ or ─CF_3_ groups) extends molecular length and promotes charge delocalization, thereby reducing anion electronegativity and potentially increasing ACN.^[^ ^14^, ^26^ ^]^ However, excessive fluorination may hinder Li^+^ mobility due to stronger anion–Li^+^ interactions.^[^ ^16a^ ^]^ The aN descriptor reflects the proportion of imide‐type anions (e.g., FSI^−^ and TFSI^−^). While imide anions offer favorable charge delocalization, an excessive aN value can result in overly strong Li^+^–anion binding, adversely affecting ion transport kinetics.^[^ ^14^, ^16^ ^]^

The recommended range for c_Li_+ lies between 0.35 and 0.65, corresponding to Li: O molar ratios from ≈1:5.1 to 1:3.0. This range falls within the medium to high concentration regime, consistent with prior reports suggesting enhanced SEI formation and ionic transport in this range.^[^ ^6^, ^7^, ^14^, ^27^ ^]^ In contrast, the optimal range for InOr lies at the higher end of the dataset distribution, indicating that inorganic‐rich anion structures are more favorable for achieving the desired coordination environment. The F/O ratio is recommended to fall between 0.05 and 0.25, suggesting that salts with higher fluorine content and lower oxygen content should be prioritized. This aligns with previous studies showing that increasing fluorine substitution can weaken solvent–Li^+^ coordination, thereby promoting Li^+^ conductivity and reducing SEI reactivity.^[^ ^14^, ^28^ ^]^

### 2.4 Optimal Composition Design and Validation

Guided by the machine‐learning‐derived optimal feature space, eleven electrolyte formulations were rationally constructed by combinatorially pairing five lithium salts with eight ether‐based solvents, aligned with the compositional space explored in the MD simulations. The corresponding feature distributions of these formulations are visualized in Figure 4d, with detailed compositions listed in Table S5, Supporting Information. It is worth noting that not all formulations strictly fall within the optimal range of every feature. This deviation arises from the discrete nature of salt–solvent chemistry and the feature‐wise independence assumed in the ML model. Consequently, the “optimal region” should be interpreted as a directional design space, offering practical guidance rather than rigid constraints (see Section 2.3).

Among the eleven candidates, four exhibited phase instability, such as freezing or stratification (Figure S35, Supporting Information), reflecting real‐world limitations absent in simulation assumptions. Specifically, the MD models i) assumed full miscibility of all salt–solvent combinations and ii) treated feature values as continuously tunable across component combinations. In contrast, real electrolyte systems are constrained by solubility limits and discrete component compatibility, which often prevent realization of idealized formulations.

**Figure 5:**
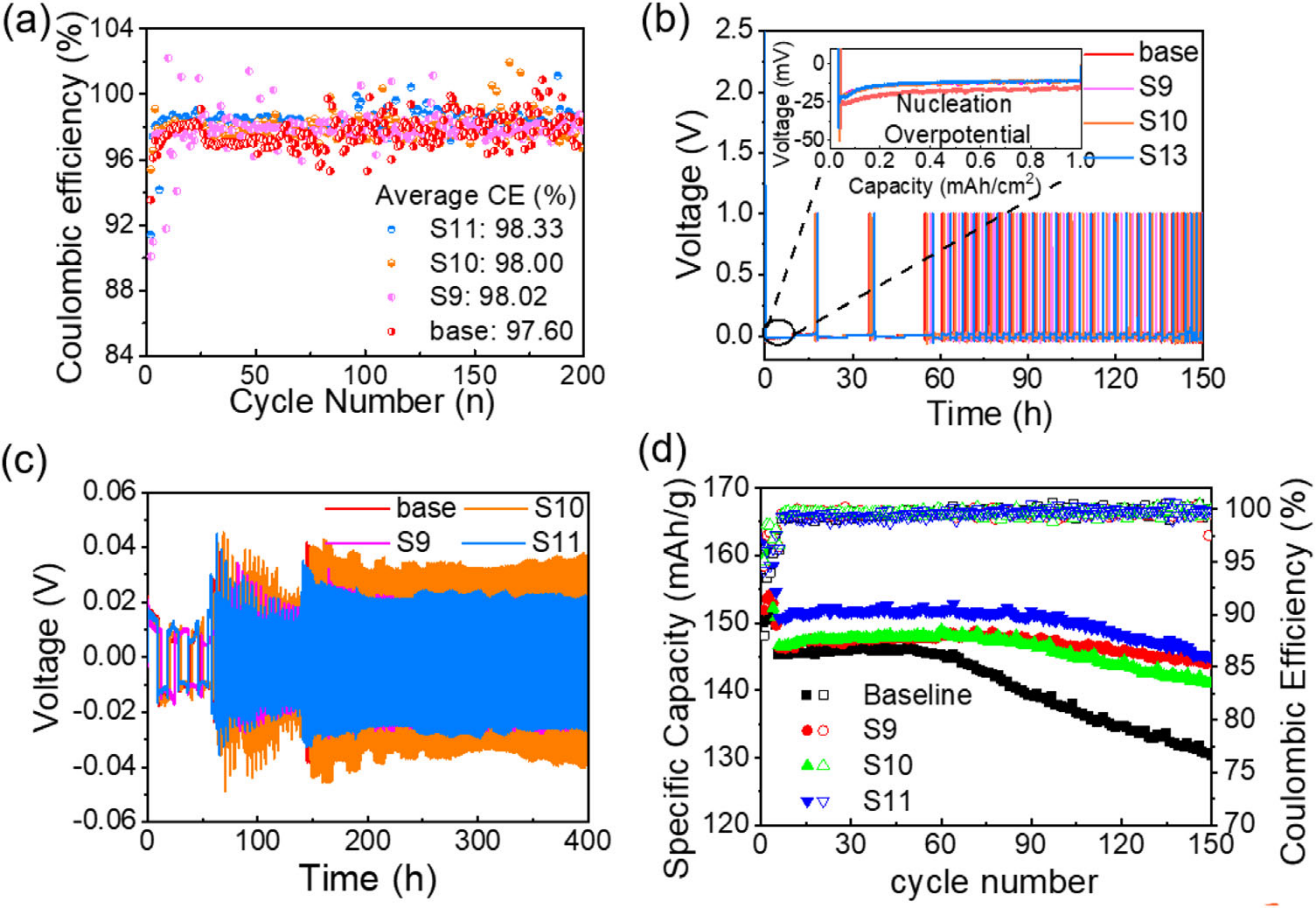
Electrochemical performance evaluation of four electrolyte formulations. a) Coulombic efficiency (CE) profiles of Li||Cu half‐cells cycled at 1 mA cm^−2^ with a plating capacity of 1 mAh cm^−2^. b) Corresponding voltage profiles highlighting nucleation and polarization overpotentials. c) Long‐term galvanostatic cycling of symmetric Li||Li cells at 1 mA cm^−2^, demonstrating interfacial stability. d) Full‐cell cycling performance of Li||LFP cells at 1C after formation cycles (0.1C/0.33C/0.5C), comparing Baseline, S9, S10, and S11 electrolytes.

CE measurements were carried out for the remaining seven stable formulations, using LiFSI–DME (Li:O = 1:4) as the reference. Among them, S9, S10, and S11 exhibited significantly improved CE values, with S11 achieving the highest (98.33%), followed by S10 (98.00%) and S9 (98.02%), all outperforming the baseline (97.60%) (Figure 5a). These trends were corroborated by Aurbach‐type CE tests (Figure S36, Supporting Information), which confirmed consistent performance rankings across methodologies. Voltage profiles of Li||Cu cells (**Figure** **5**b) revealed that S11 significantly reduced polarization overpotential, albeit at the cost of a slightly increased nucleation overpotential (31 mV vs 24 mV for the baseline). While S9 and S10 also reduced polarization overpotential, their higher nucleation barriers (≈39 mV) may reflect less favorable SEI formation. Symmetric Li||Li cycling data (Figure 5c; Figures S37–S43, Supporting Information) further confirmed that S11 exhibited the lowest and most stable overpotential, indicating superior interfacial stability. Full‐cell tests with Li||LFP (Figure 5d; Figure S44, Supporting Information) demonstrated excellent electrochemical stability of the optimized electrolytes. In contrast to the baseline (initial CE = 89.26%, capacity fade after 50 cycles), S9–S11 delivered higher initial Coulombic efficiencies (>94%) and sustained stable capacity retention for over 150 cycles. Among them, S11 consistently outperformed others across all metrics. These results indicate that, although the ML model was initially developed to optimize Li‐metal anode compatibility, the identified formulations also exhibited strong full‐cell performance with commercial LFP cathodes. This highlights the framework's broader applicability and supports future integration of cathode‐specific constraints into model development.

Feature analysis of these top‐performing electrolytes (Figure S45, Supporting Information) revealed that optimal performance is associated with moderate increases in *E*_b_, aC, and sC, alongside controlled reductions in aO, sO, and aN. Simultaneous maximization of aF, F/O, and InOr further contributed to performance gains. Among solvents, DEE outperformed DME due to its favorable positioning in terms of *E*_b,_^[^ ^14^, ^29^ ^]^ sO, and sC. A Li:O molar ratio of 1:4 was selected to balance full salt dissolution with effective Li⁺ solvation and enhanced ACN. Addition of LiNFS improved aF and F/O; while, maintaining low aO and aN, which collectively enabled the exceptional performance observed for S11.

Collectively, these findings underscore the power of machine‐learning‐guided trend analysis and optimal region construction in accelerating rational electrolyte design. The framework not only captures key structure–property correlations—such as *E*_b_ and elemental balance (O, F, C, N)—but also demonstrates predictive validity through strong agreement with experimental outcomes in both half‐cell and full‐cell configurations. This integrative approach provides a scalable, data‐driven strategy for discovering next‐generation electrolytes tailored for diverse battery chemistries.

## 3 Conclusion 

In this study, MD simulations were employed to systematically investigate the solvation structures of 166 electrolyte formulations, derived from combinations of five lithium salts and eight ether‐based solvents. The results revealed distinct trends: the ACN increased with salt concentration, whereas both the SCN and *D*_Li_+ decreased. In addition, variations in solvated cluster morphologies were observed across different salt–solvent pairs, even at identical concentrations, highlighting the complexity of coordination environments in mixed‐component systems.

Subsequent feature selection identified eleven molecular descriptors relevant to electrolyte performance. Among them, *E*_b_, sO, and aO emerged as common high‐importance features for ACN, SCN, and *D*_Li_+. These descriptors govern Li⁺ solvation strength and coordination geometry, potentially driving the formation of distinct solvation aggregates such as AGGs, CIPs, and SSIPs.

Using the selected features, three ML datasets were constructed for ACN, SCN, and *D*_Li_+, respectively. Multiple algorithms were benchmarked, and GPR was identified as the most effective model. The trained GPR models were used for predictive screening, followed by statistical analysis of high‐performing outputs to define an optimal composition region, characterized by favorable coordination behavior and transport properties.

Based on this ML‐guided design space, eleven electrolyte formulations were proposed, and seven stable systems were experimentally validated. Among them, a formulation comprising DEE as the solvent, a Li:O molar ratio of 1:4, and LiNFS as an additive demonstrated superior performance in LMBs, achieving high Coulombic efficiency, low polarization overpotentials, and excellent cycling stability under 1C operation.

Beyond validation, this work demonstrates the practical utility and scalability of the developed ML framework. The model can be readily retrained with new experimental or computational data, making it a flexible tool for accelerating the exploration of vast electrolyte design spaces. This integrative strategy provides a foundation for data‐driven discovery of next‐generation electrolytes across diverse battery chemistries.

## 4 Methodology/Calculation

### 4.1 Molecular Dynamics Simulation

Initial ground‐state geometries of individual molecules, including bond lengths, angles, and dihedral parameters, were optimized using DFT with the B3LYP exchange‐correlation functional. Binding energies (*E*_b_) were calculated following established DFT protocols consistent with prior reports.^[^ ^14j^ ^]^ For each single‐salt‐single‐solvent system, representative molecular models with box dimensions of 5 nm were constructed using the Moltemplate toolkit, and atom counts were scaled according to stoichiometrically determined concentration ratios.

MD simulations were performed using the LAMMPS package (version 3Mar2020),^[^ ^30^ ^]^ with detailed simulation parameters provided in the Supporting Information S1. After energy minimization and equilibration steps, produced trajectories were collected and analyzed to extract radial distribution functions (RDFs), coordination numbers, and *D*_Li⁺_, following validated approaches for solvation structure characterization.^[^ ^16^, ^31^ ^]^

With respect to concentration selection strategy, electrolyte concentrations were systematically designed based on the spatial coordination theory of Li^+^ ions (Figure S46, Supporting Information),^[^ ^17^ ^]^ which defines preferred solvation structures using O‐donor ligands from both anions and solvent molecules.^[^ ^6^, ^14^, ^25^ ^]^ To span a broad spectrum of coordination environments, simulations were conducted over a series of Li^+^:O molar ratios, including 1: 2, 1: 4, 1: 5, 1: 6, and 1: 8, and extending to dilute limits up to 1: 40. These ratios corresponded to Li^+^ concentrations ranging from highly concentrated to dilute regimes. The complete list of concentrations (in mol L^−1^) for all salt–solvent combinations is provided in the Supporting Information S2.

### 4.2 Machine Learning Model Development

The anion coordination number (ACN) and solvent coordination number (SCN) are two critical descriptors influencing SEI formation. High ACN values generally promote the formation of thin, inorganic‐rich, and mechanically robust SEI layers; while, elevated SCNs tend to favor thick, organic‐dominated interphases.^[^ ^16^ ^]^ To strategically modulate these parameters, recent studies have employed i) highly concentrated or super‐concentrated electrolytes and ii) weakly coordinating solvents.^[^ ^7^, ^9^, ^10^, ^14^, ^16^, ^25^, ^28^ ^]^ Accordingly, the ACN and SCN values derived from our MD simulations were adopted as primary labels for ML modeling.

In parallel, *D*_Li_+ was chosen as a third target label. While essential for minimizing interfacial resistance and ensuring rapid charge transport, *D*_Li_+ often exhibits a trade‐off with electrolyte concentration—higher lithium concentrations increase viscosity, thereby compromising ion mobility and electrode wettability.^[^ ^25^, ^32^ ^]^ Thus, *D*_Li_+ was incorporated to balance interfacial stability against bulk transport properties in the model.

#### 4.2.1 Feature Engineering and Normalization

With reference to feature selection and construction, eight compositional features were adopted from prior literature,^[^ ^11^ ^]^ representing molar fractions of key elements: aC, aO, aN, and aF (from anions), sC and sO (from solvents), the F/O atomic ratio, and the inorganic‐to‐organic ratio (InOr, excluding H and Li). To capture electronic and solvation‐related effects, three additional physicochemical features were introduced: 1) Solvent dipole moment (*µ*): indicating polarity and affecting solvation energy; 2) *E*_b_: influencing lithium desolvation dynamics and SEI formation;^[^ ^14^, ^33^ ^]^ and 3) lithium salt concentration (*c*_Li⁺_): governing ion‐pairing equilibria (aggregates [AGGs], contact ion pairs [CIPs], and solvent‐separated ion pairs [SSIPs])^[^ ^34^ ^]^ and modulating HOMO–LUMO energy levels in ether solvents.^[^ ^7^, ^14^, ^16^, ^35^ ^]^

#### 4.2.2 Feature Selection

Two complementary feature selection strategies were applied: i) Correlation‐based selection: A five‐round stochastic subsampling procedure was performed, where 80% of the dataset was randomly selected in each round to compute Pearson correlation coefficients. The top eight features from each round were recorded, and the most frequently selected features across all rounds (from a pool of 11 recurring features) were designated as the final subset. ii) Recursive feature elimination (RFE): An iterative backward elimination approach was applied to a baseline model trained on the full feature set. In each round, the least important feature—quantified via four distinct importance metrics: mutual information coefficient (MIC), *R* ^2^ score, information gain, and ΔMSE—was removed. Five repetitions of fivefold cross‐validation (5 × 5 CV) were used to ensure model robustness. Final rankings were determined by aggregating scores across all folds and metrics. Features with aggregate rankings above the median were retained as optimal subsets for each of the three target labels (ACN, SCN, and *D*_Li_+). Complete methodological details, including equations and workflows, are provided in the Supporting Information S1 (Figure S47 and Equations (S2)–(S7), Supporting Information).^[^ ^36^ ^]^

#### 4.2.3 Model Selection and Evaluation

Three machine learning algorithms—Extreme Gradient Boosting (XGBoost, XGB), Random Forest (RF), and GPR—were benchmarked using three evaluation metrics: coefficient of determination (*R* ^2^), root mean squared error (RMSE), and correlation coefficient. Each model was trained on the optimized feature subsets and evaluated via tenfold cross‐validation for each of the three target properties (ACN, SCN, and *D*_Li_+). The model achieving the best trade‐off—that is, maximizing *R* ^2^ and correlation while minimizing RMSE—was selected as the final architecture for downstream prediction. In this study, GPR emerged as the optimal model and was used for further screening.

#### 4.2.4 Predictive Screening and Optimal Trend Extraction

For each target property (ACN, SCN, and *D*_Li_+), independent GPR models were trained using the selected feature subsets. To prevent convergence to local optima during training, an ensemble Bayesian optimization (EBO) strategy adapted from previous work^[^ ^37^ ^]^ was employed. Only models with validation *R* ^2^ > 0.65 were retained for further prediction.

The normalized output space (0–1) of each model was discretized into 20 candidate values per feature, with 10 values randomly sampled in each round. A total of 10 independent optimization cycles were executed for each prediction set, and in each cycle, the top 60% of predicted solutions was retained. The final “optimal region” was defined by the intersection of high‐performing solutions across all three properties (i.e., ACN ∩ SCN ∩ *D*_Li_+).

Statistical analysis of feature distributions within this region was conducted using frequency histograms. The most frequently occurring value range (modal bin) for each feature was designated as the ML‐recommended design range. These collectively defined the global optimal subspace, which informed the trend analysis and experimental design.

#### 4.2.5 Statistical Analysis

To ensure consistent data handling and improve machine learning model robustness, statistical processing was applied as follows:

##### Data Pre‐Processing

All input descriptors were normalized using min–max scaling to eliminate dimensional bias across features during training and feature selection:

(1) $$V_{\mathrm{norm}}=\left(V_{x}-V_{\min}\right)/\left(V_{\max}-V_{\min}\right)$$

where *V*_norm_, *V*_min_, and *V*_max_ are the normalized, minimum, and maximum values of one feature set.

In addition, the *D*_Li_+ logarithmically transformed to stabilize model convergence: A logarithmic transformation was applied to improve ML model training stability, as described by Equation (2):

(2) $$V_{\mathrm{diff}}=-\mathrm{lg}\left(V_{\mathrm{diff}\_\mathrm{original}}\right)$$

where *V*_diff_ is the converted feature value and *V*_diff_original_ is the original value calculated by the MD simulation. This transformation helps improve model fitting accuracy for values spanning multiple orders of magnitude.

##### Sample Size

For ML model construction and validation, each target label (ACN, SCN, and *D*_Li_+) was associated with a dataset consisting of 166 samples. The number of selected input features was six for ACN, seven for SCN, and six for *D*_Li_+, based on prior feature selection using multi‐metric importance evaluation.

##### Statistical Methods

Model performance was assessed using tenfold cross‐validation, with repeated training/testing splits to ensure reproducibility and minimize overfitting. Key evaluation metrics included: *R* ^2^ (coefficient of determination), root mean square error (RMSE), and Pearson correlation coefficient. For electrochemical data comparisons, no hypothesis testing was applied as emphasis was placed on trend validation and relative performance improvement across electrolyte formulations.

##### Software Used

All statistical analyses and machine learning workflows were implemented using Python (v3.10) with the scikit‐learn library (v0.24.2), and data visualization was conducted using Matplotlib and Seaborn packages.

## 5 Experimental Section

### Materials

All lithium salts and solvents were purchased from MACKLIN and used without further purification. The following reagents were employed: Lithium bis(fluorosulfonyl)imide (LiFSI): 99.8%, CAS 171611‐11‐3, Lithium bis(trifluoromethanesulfonyl)imide (LiTFSI): 99.9%, CAS 90076‐65‐6, Lithium nonafluorobutanesulfonate (LiNFS): 95%, CAS 131651‐65‐5, Lithium trifluoroacetate (LiTFA): 99.5%, CAS 33454‐82‐9, Lithium difluoro(oxalato)borate (LiDFOB): 99%, CAS 409071‐16‐5, 1,2‐Dimethoxyethane (DME): 99.95%, CAS 110‐71‐4, Diethyl ether (DEE): 99%, CAS 629‐14‐1, Diisopropyl ether (DIPE): 99%, CAS 108‐20‐3 (used in place of dipropyl ether due to similar binding energy and dipole moment, Figure S48, Supporting Information), Triethylene glycol dimethyl ether (TREGDME): 99%, CAS 112‐49‐2.

Electrolyte solutions were prepared in an argon‐filled glove box (H₂O < 0.1 ppm, O₂ < 0.1 ppm) at 23 °C. Lithium salts were dissolved into solvents at predetermined molar ratios under continuous magnetic stirring (500 rpm, 12 h). Anodes consisted of lithium foil (500 µm thickness, *Ø*15.6 mm; China Energy Lithium Co., Ltd.) and cathodes were fabricated from copper foils (12 µm thickness, *Ø*16 mm; Shenzhen Londian Wason Holdings Group Co., Ltd.). LiFePO_4_ (LFP, Nanjing Lithium Source Nano Technology Co., Ltd) cathode was prepared with active material loadings of ≈2 mg cm^−2^. The electrode slurry was composed of active material, Super P, and PVDF in a weight ratio of 8:1:1.

### Materials Characterization

Electrolyte density was measured using a KORRIZAI digital densitometer. The instrument was submerged in 50 mL of electrolyte, and measurements were recorded after stabilization (no visible bubble adhesion). Raman spectra were acquired using a Horiba LabRAM HR evolution spectrometer equipped with a 785 nm laser source. Measurements were conducted with a 2 µm laser spot size, 1.5 mW laser power, 30 s acquisition time, and a spectral resolution of 1 cm^−1^. Fourier transform infrared spectroscopy (FTIR) analysis was conducted using a Thermo Fisher Nicolet iS5 spectrometer to assess molecular vibrations and bonding interactions.

### Electrochemical Measurements

All electrochemical measurements were conducted using CR2032‐type coin cells assembled in an argon‐filled glove box. Polyethylene separators (16 µm thickness, *Ø*19 mm) were used, with 120 µL of electrolyte per cell. Symmetric Li||Li cells employed two lithium foils (500 µm thick) as electrodes. Li||Cu half‐cells used a copper disk (*Ø*16 mm) as the working electrode and lithium foil as the counter/reference. All testing was performed on a LAND battery tester at 25 ± 0.5  °C.

The area capacity was fixed at 1 mAh cm^−2^. For CE tests, Li was plated onto the Cu substrate at 0.1 mA cm^−2^ for three cycles to stabilize the interface. Subsequent galvanostatic cycles included: 1 cycle at 0.33 mA cm^−2^, 20 cycles at 0.5 mA cm^−2^, and continuous cycles at 1 mA cm^−2^. Average CE was calculated from the 51st to 150th cycle in triplicate Li||Cu cells per electrolyte formulation.^[^ ^21^, ^38^ ^]^ For Aurbach‐type CE measurements,^[^ ^39^ ^]^ the procedure included: initial Li plating (1 mAh cm^−2^ at 0.1 mA cm^−2^) followed by stripping to 1 V; formation of a Li reservoir via additional plating (1 mAh cm^−2^); and repeated plating/stripping (1 mAh cm^−2^ at 1 mA cm^−2^) until the cutoff voltage of 1.5 V was reached.

Full‐cell performance (Li||LFP) was tested over voltage ranges of 2.5–3.65 V. Formation cycles consisted of three cycles at 0.1 C, one cycle at 0.33 C, and one cycle at 0.5 C, followed by long‐term cycling at 1 C.

## Conflict of Interest

The authors declare no conflict of interest.

## Supporting information



Supporting Information

Supporting Information

## Data Availability

The data that support the findings of this study are available in the supplementary material of this article.
