# Supplementary material for: Integrating Molecular Dynamics and Machine Learning for Solvation‐Guided Electrolyte Optimization in Lithium Metal Batteries
Source: Adv Sci (Weinh). 2025 Jun 30;12(36):e04997. doi: 10.1002/advs.202504997 (PMC12462972; doi:10.1002/advs.202504997)
Supplement: Supplementary file 1 — Supporting Information [file ADVS-12-e04997-s001.docx]

Supplementary Material-1 for

## Integrating Molecular Dynamics and Machine Learning for Solvation-Guided Electrolyte Optimization in Lithium Metal Batteries

*Xiwang Chang^1,3,^^[[1]](#footnote-0)^a, Yang Yang^2,a^, Weiheng Xu^2^, Zhe Wang^3^, Wenhan Li^3^, Hongda Gao^3^,* *Dubin Huang^3^, Aijun Li^2,^^[[2]](#footnote-1)^*,* *Yaofeng Zhu^1,*^*

^1^ School of Materials Science & Engineering, Zhejiang Sci-Tech University, Hangzhou 310018, China

^2^ School of Materials Science and Engineering, and Academy for Advanced Interdisciplinary Studies, Peking University, Beijing, 100871, China

^3^ Zhejiang Gold Feather New Energy Technology Co., Ltd, Huzhou 313300, China


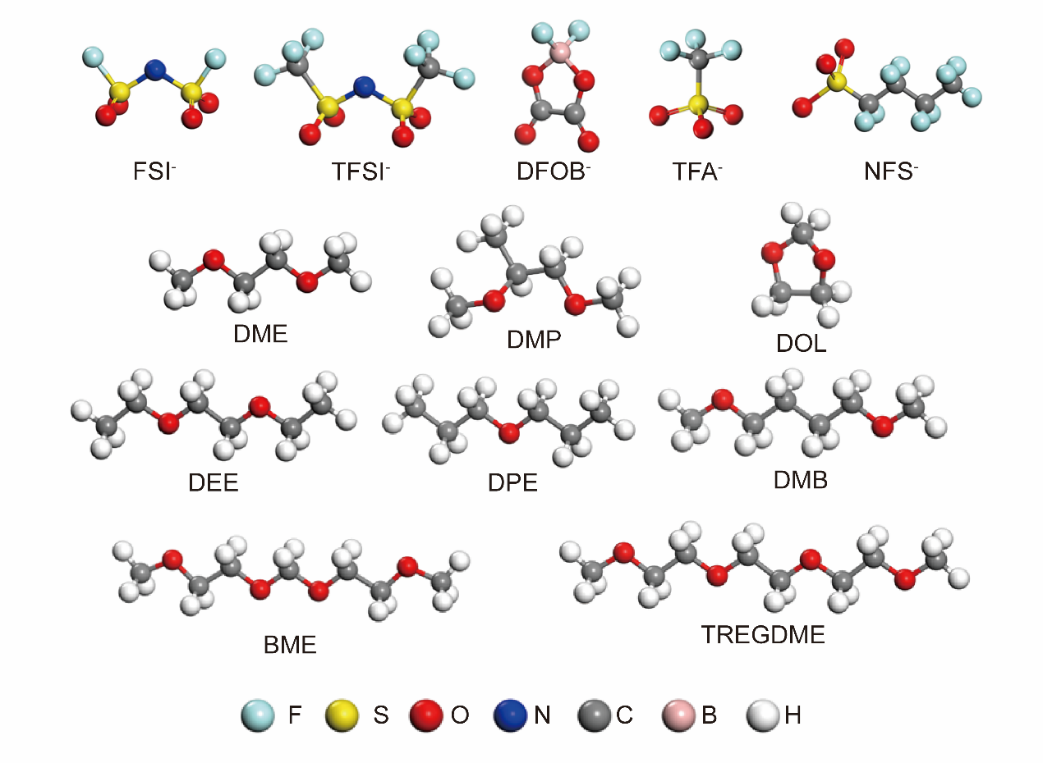


**Figure S1.** Molecular structures of five anions and eight solvents. These structures were employed as the initial state to build the solution lattice models using the Mol-template package. Abbreviated specification: DEE: 1,2-diethoxyethane; DMB: 1,4-dimethoxylbutane; TREGDME: tetraethylene glycol dimethyl ether; DPE: dipropyl ether; BME: 1-Methoxy-2-(2-methoxyethoxymethoxy)ethane; DOL: 1,3-dioxolane

**Table S1.** Electrolyte Compositions for MD Simulation

|  | LiFSI | LiTFSI | LiDFOB | LiTFA | LiNFS |
| --- | --- | --- | --- | --- | --- |
| DME | 8 | 8 | 8 | 6 | 6 |
| DMP | 8 | 6 | 6 | 6 | 6 |
| DMB | 6 | 6 | 6 | -- | -- |
| DEE | 6 | 6 | 6 | -- | -- |
| TREGDME | 6 | 6 | 6 | -- | -- |
| DPE | 6 | 6 | 4 | -- | 6 |
| BME | 6 | -- | -- | 6 | 4 |
| DOL | -- | 6 | -- | -- | -- |

**Molecular Dynamics Simulation Consequences of Rest Solutions**


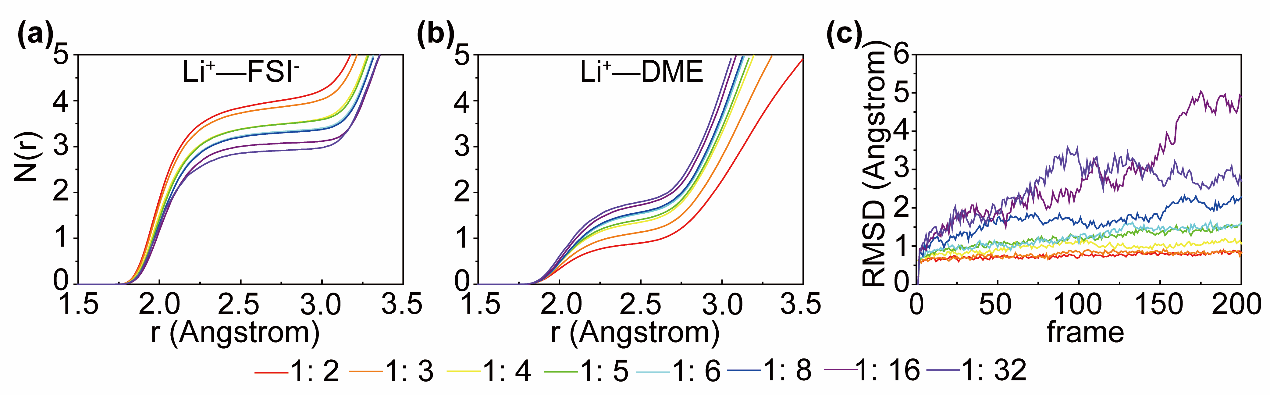


**Figure S2.** The anion coordinate number (a), solvent coordinate number (b), and RMSD (c) were calculated at different concentrations for the LiFSI-DME series, and it was the same sequence was observed for the next 25 figures.


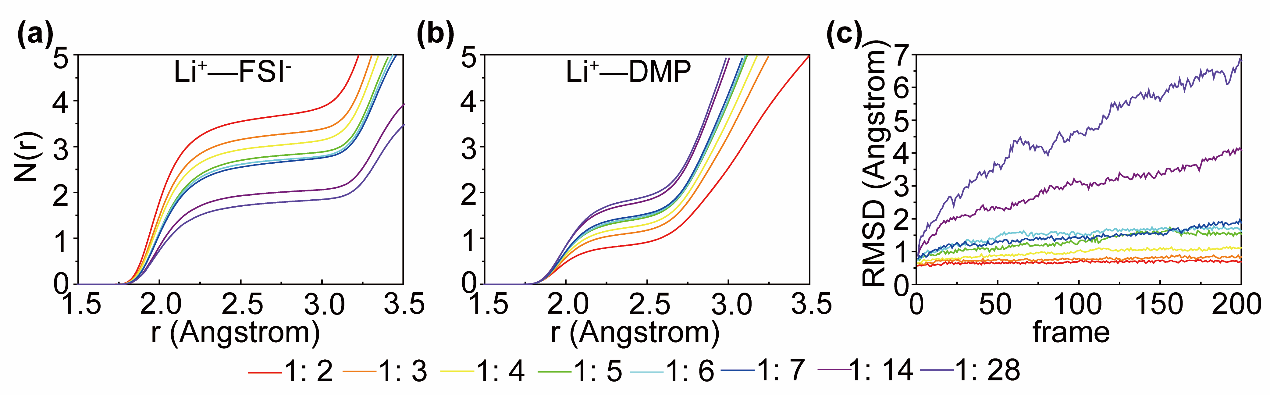


**Figure S3.** LiFSI-DMP series.


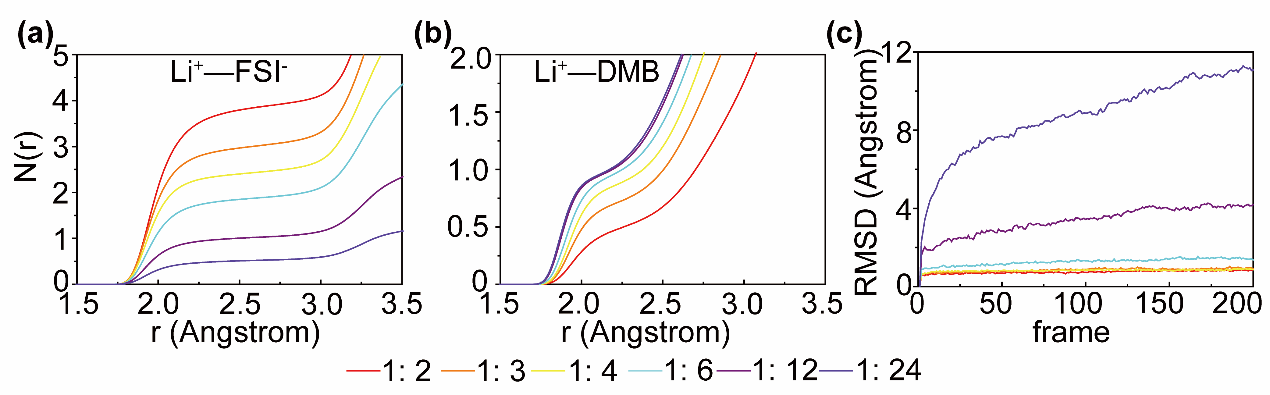


**Figure S4.** LiFSI-DMB series.


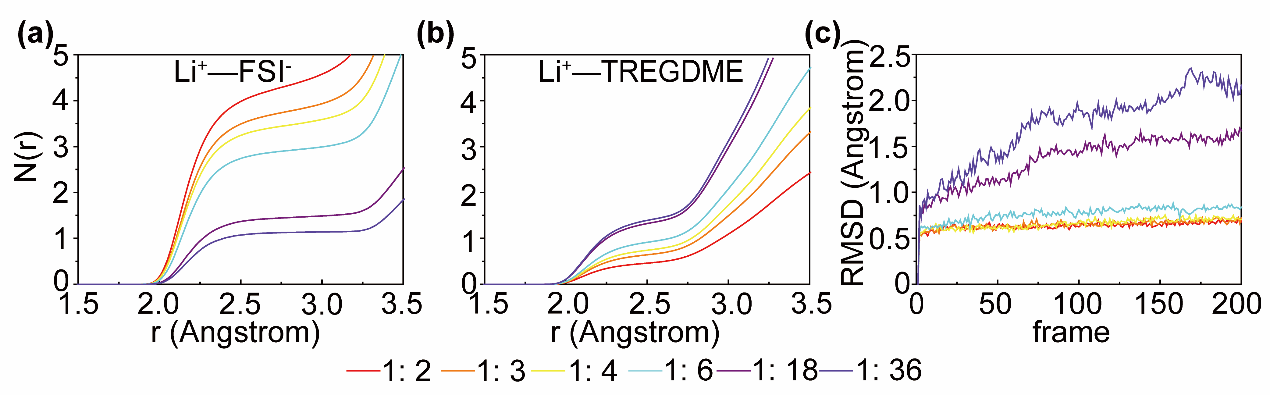


**Figure S5.** LiFSI-TREGDME series.


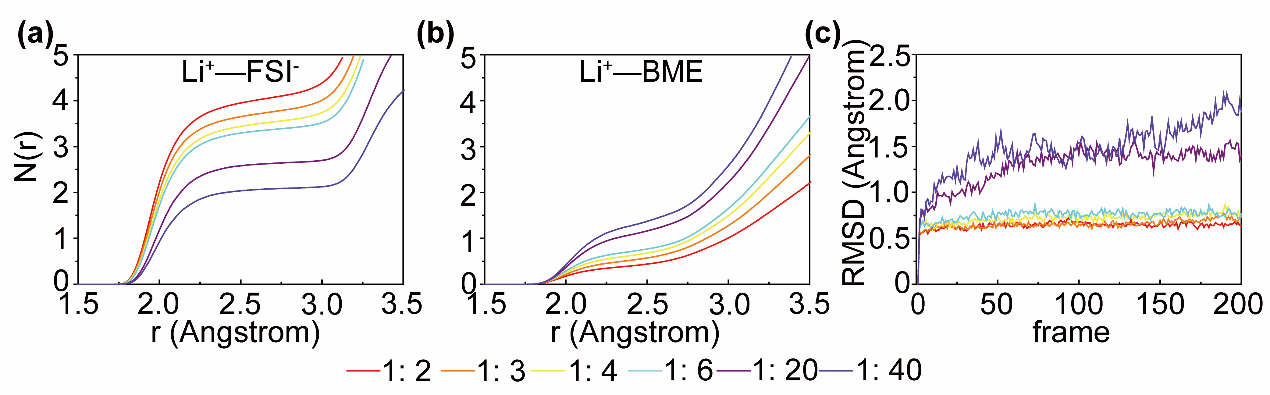


**Figure S6** LiFSI-BME series.


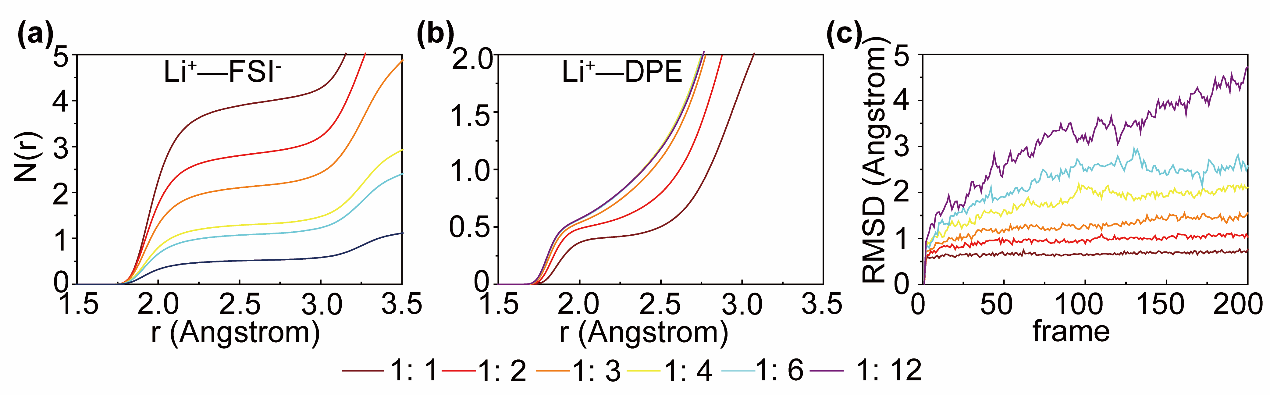


**Figure S7.** LiFSI-DPE series.


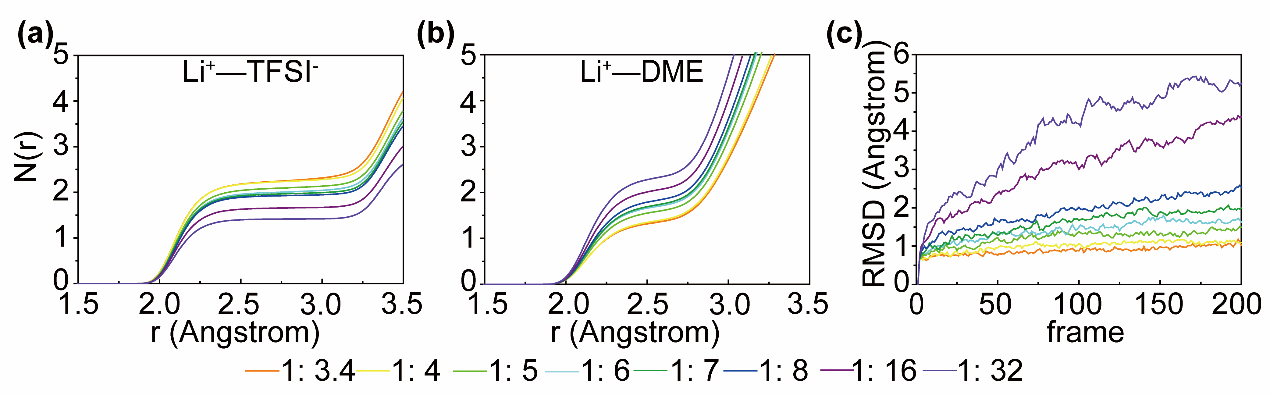


**Figure S8.** LiTFSI-DME series.


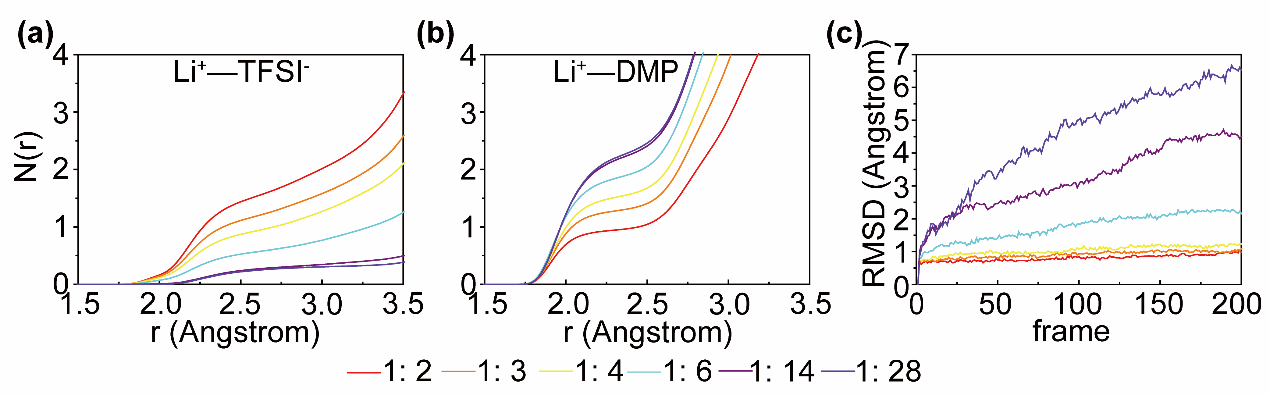


**Figure S9.** LiTFSI-DMP series.


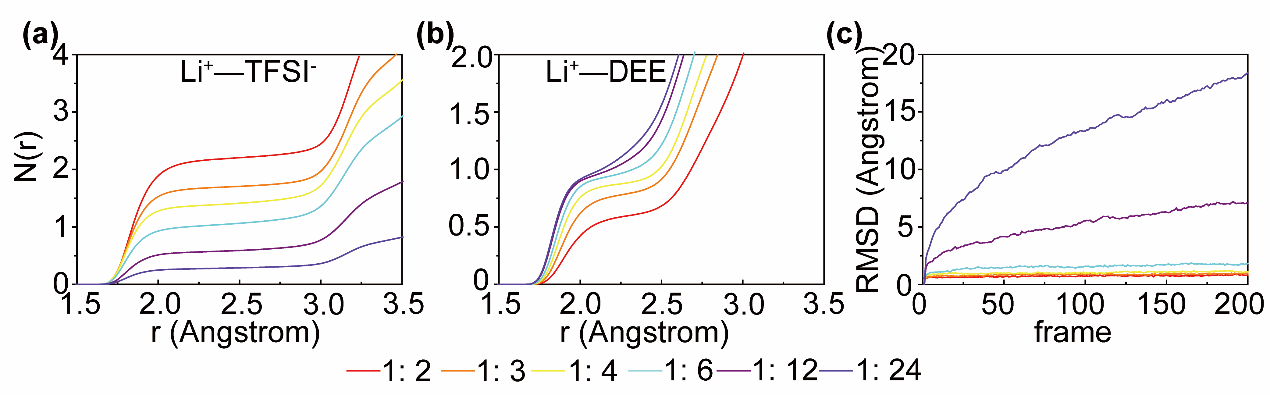


**Figure S10.** LiTFSI-DEE series.


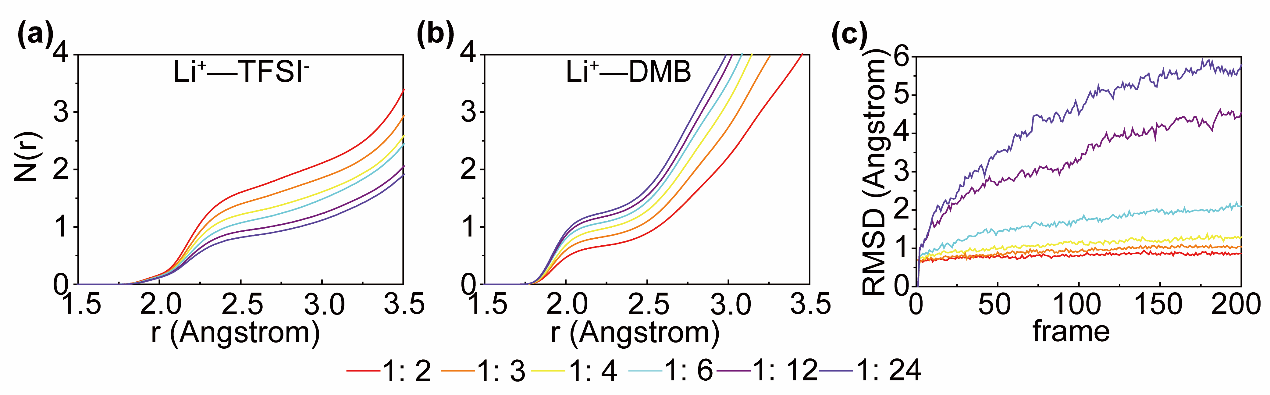


**Figure S11.** LiTFSI-DMB series.


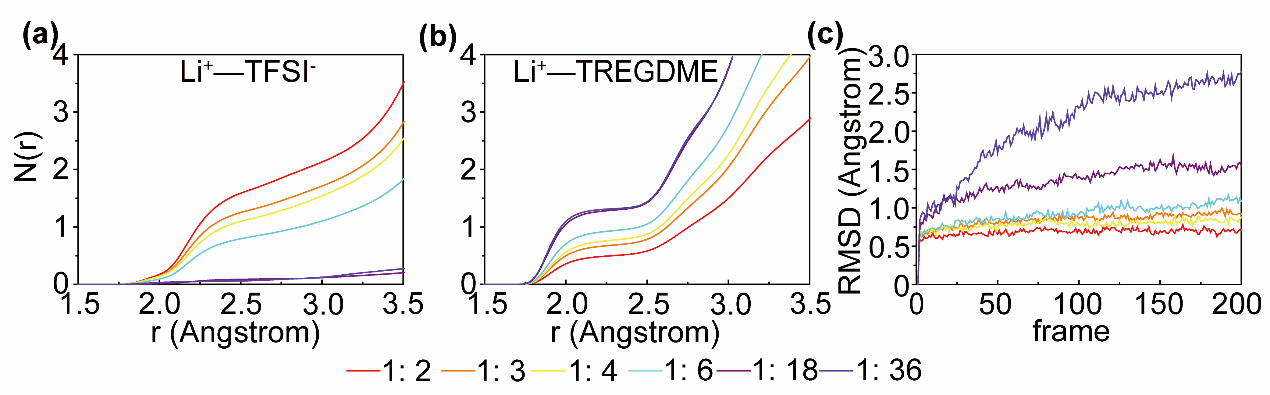


**Figure S12.** LiTFSI-TREGDME series.


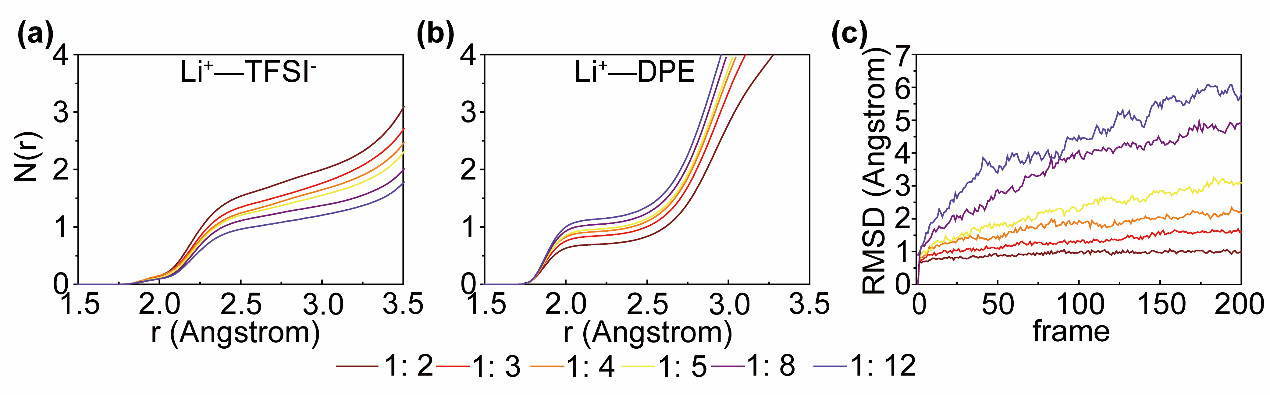


**Figure S13.** LiTFSI-DPE series.


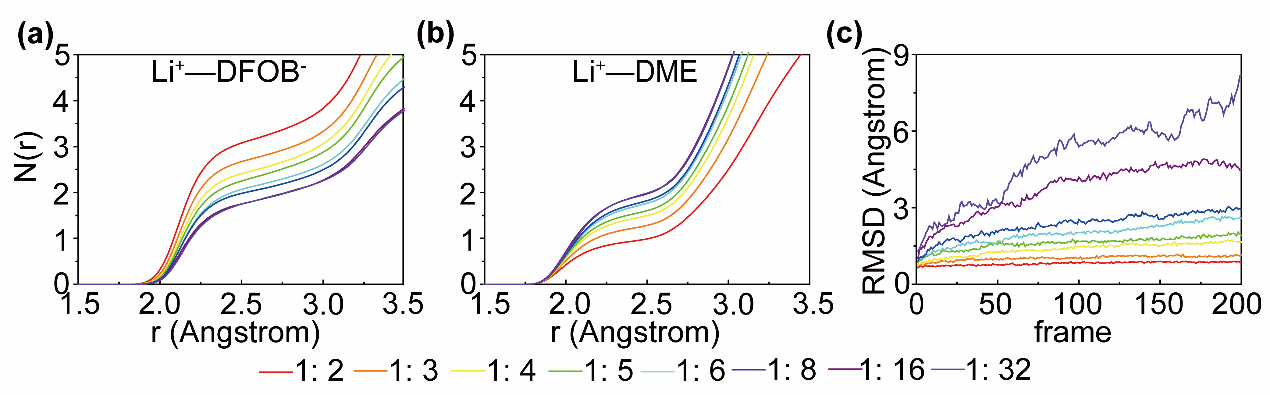


**Figure S14.** LiDFOB-DME series.


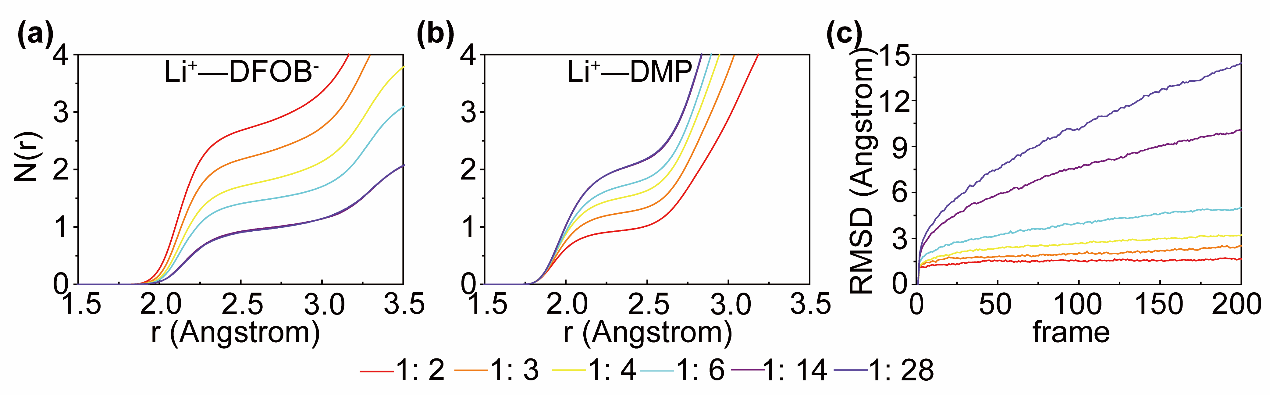


**Figure S15.** LiDFOB-DMP series.


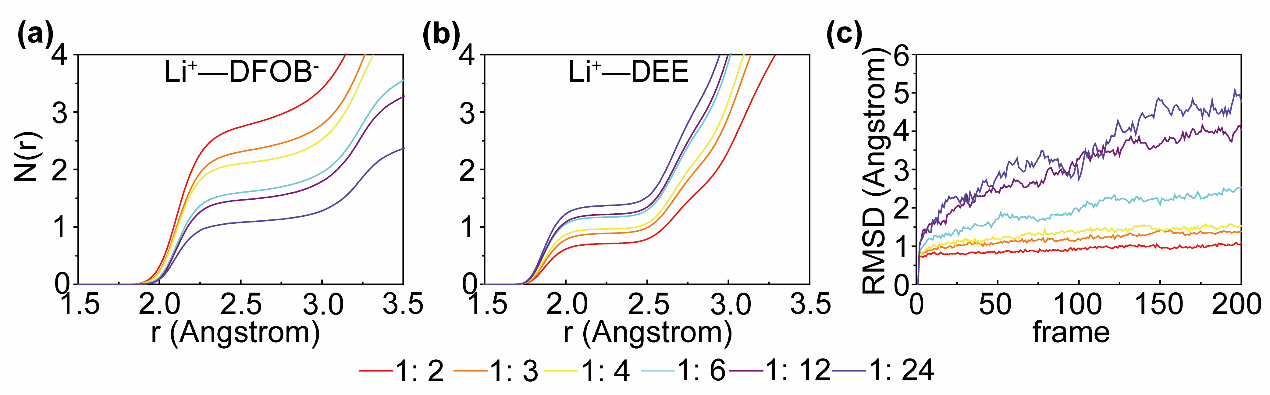


**Figure S16.** LiDFOB-DEE series.


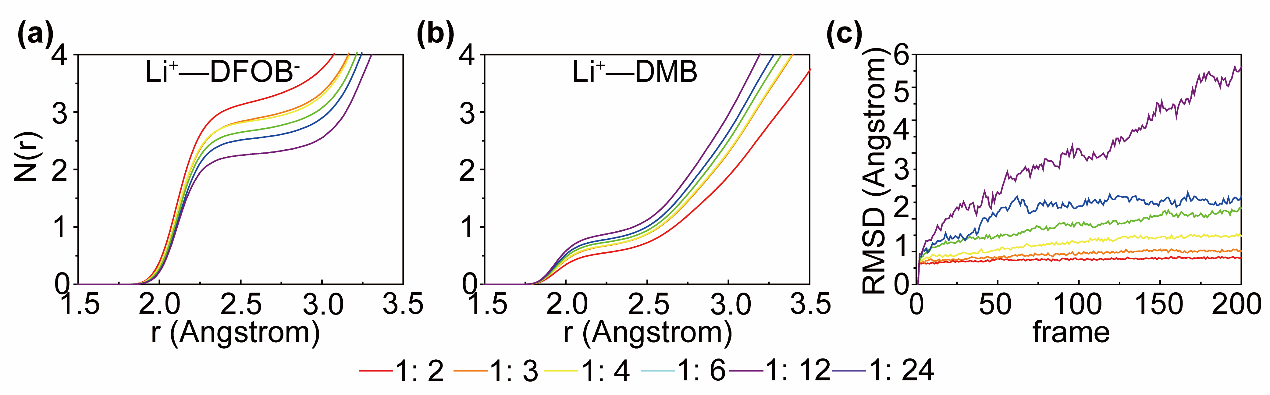


**Figure S17.** LiDFOB-DMB series.


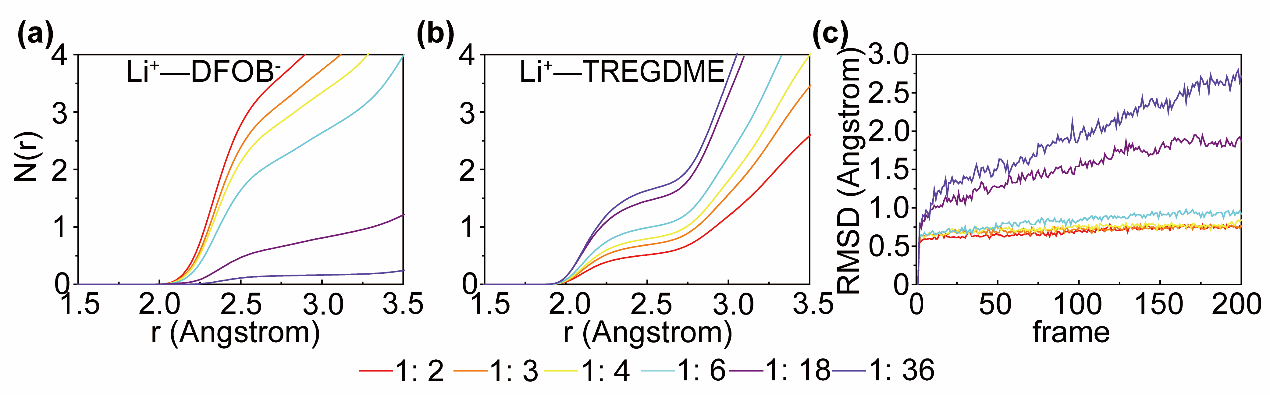


**Figure S18.** LiDFOB-TREGDME series.


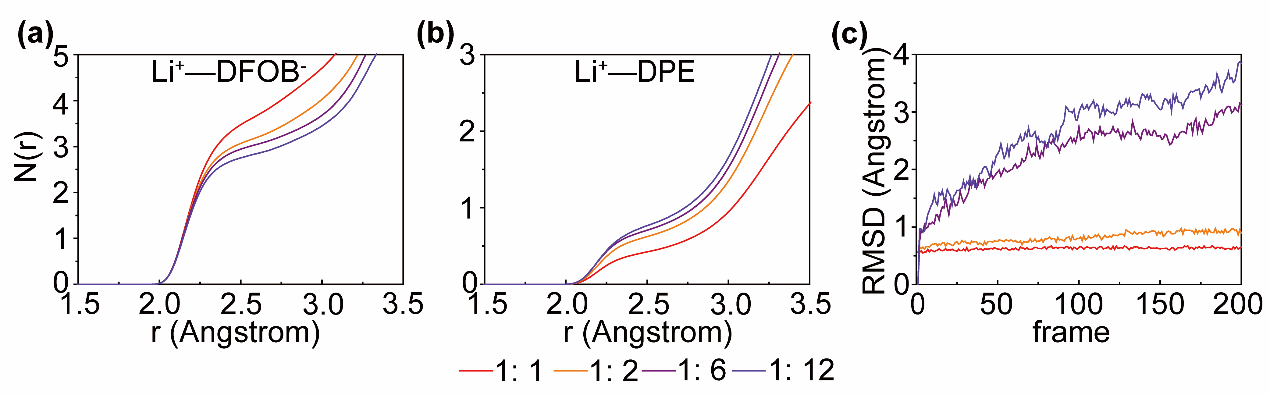


**Figure S19.** LiDFOB-DPE series.


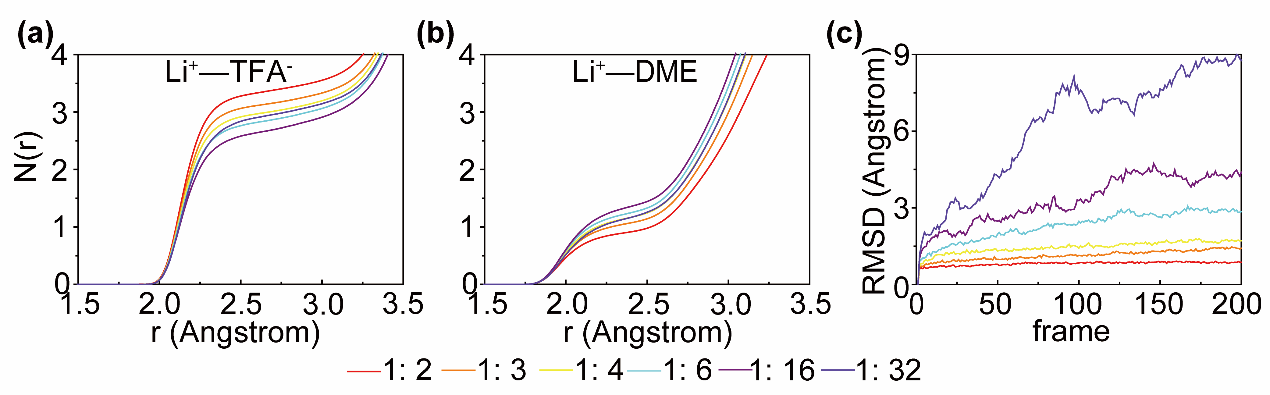


**Figure S20.** LiTFA-DME series.


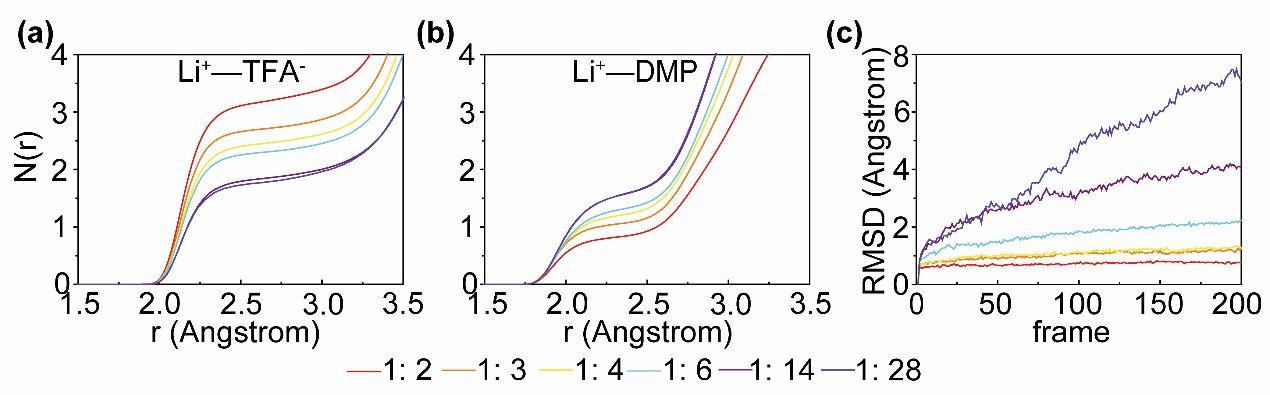


**Figure S21.** LiTFA-DMP series.


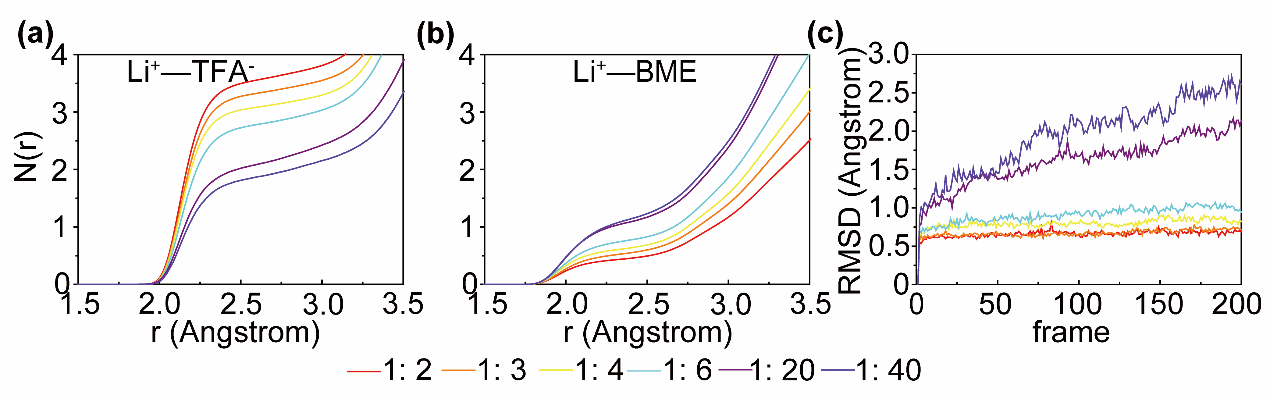


**Figure S22.** LiTFA-BME series.


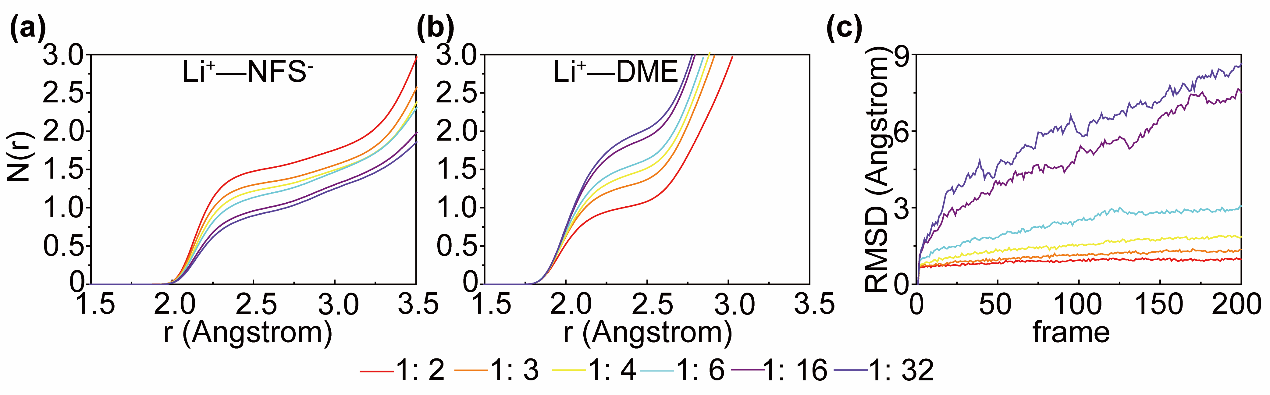


**Figure S23.** LiNFS-DME series.


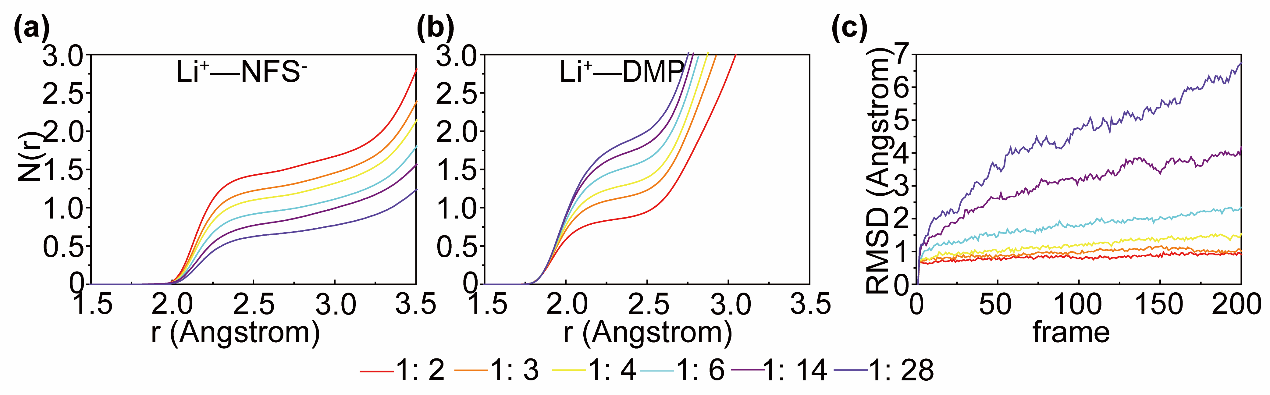


**Figure S24.** LiNFS-DMP series.


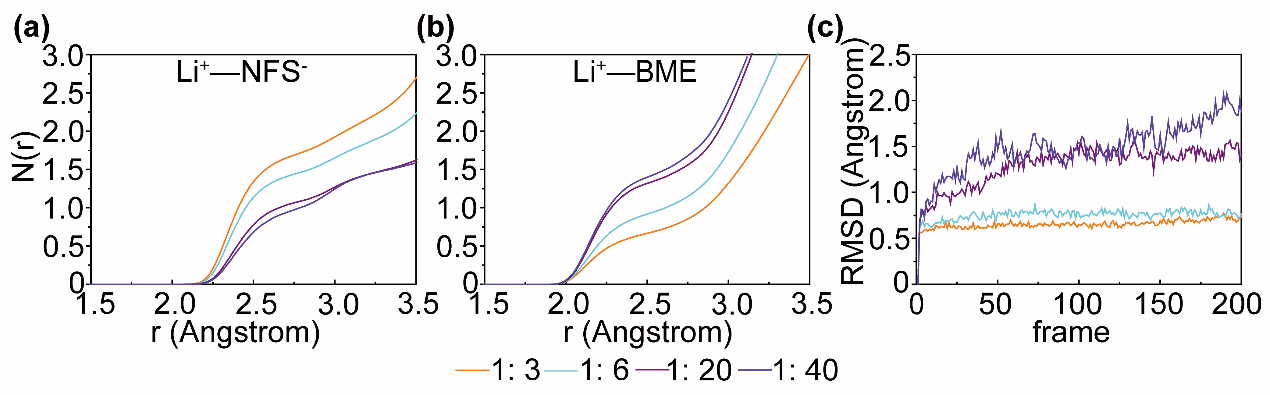


**Figure S25.** LiNFS-BME series.


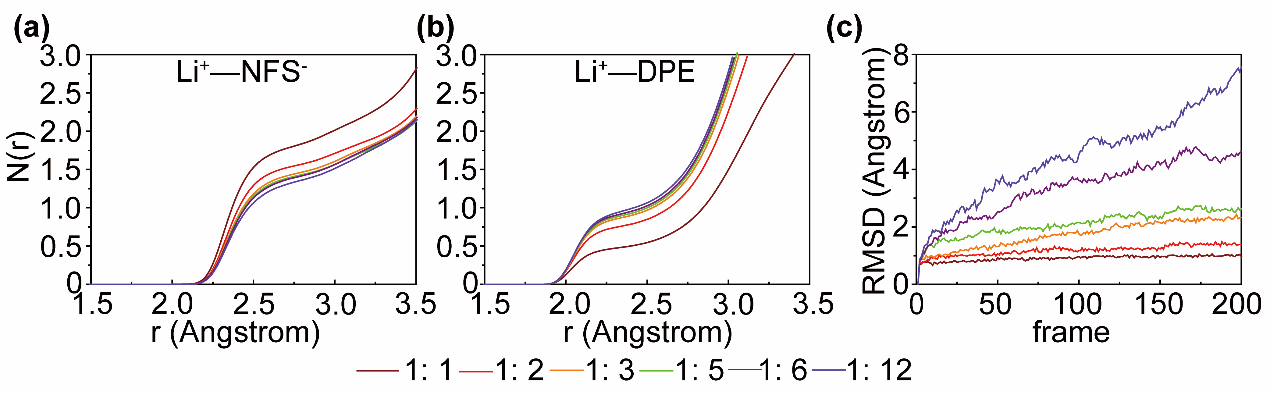


**Figure S26.** LiNFS-DPE series.


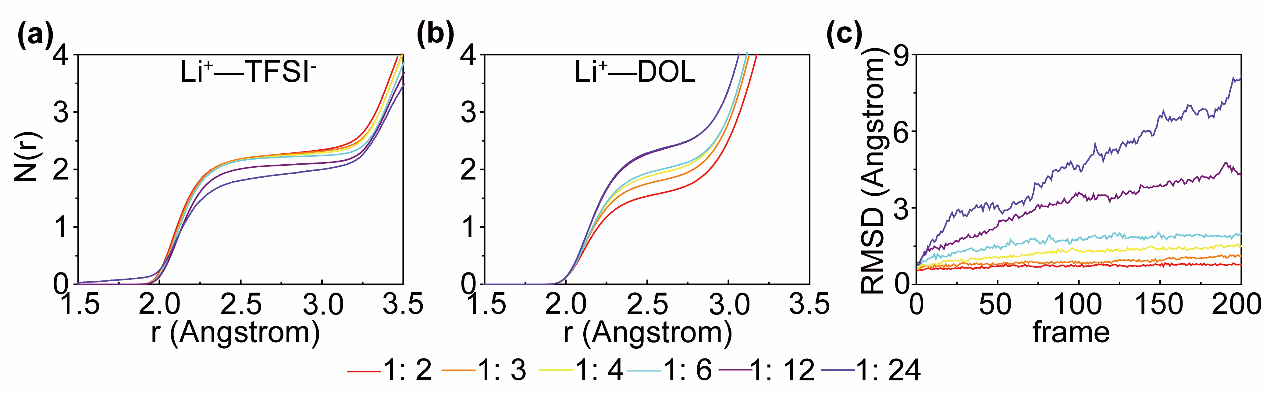


**Figure S27.** LiTFSI-DOL series.


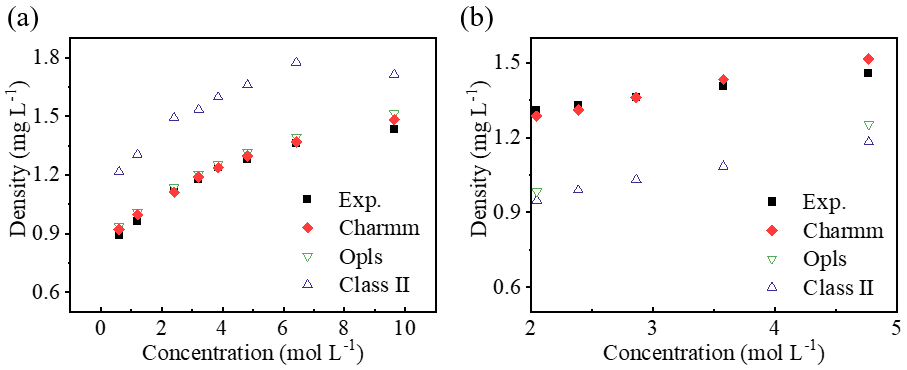


**Figure S28.** Comparison between experimental and simulated electrolyte densities using different force field parameters. (a) Density-concentration profiles of LiFSI-DME electrolytes simulated with Charmm, OPLS, and Class II force fields, compared with experimental values. (b) Simulated and experimental densities of LiTFSI-DOL electrolytes at various concentrations. Among the tested force fields, the Charmm model shows the best overall agreement with experimental data in both systems.


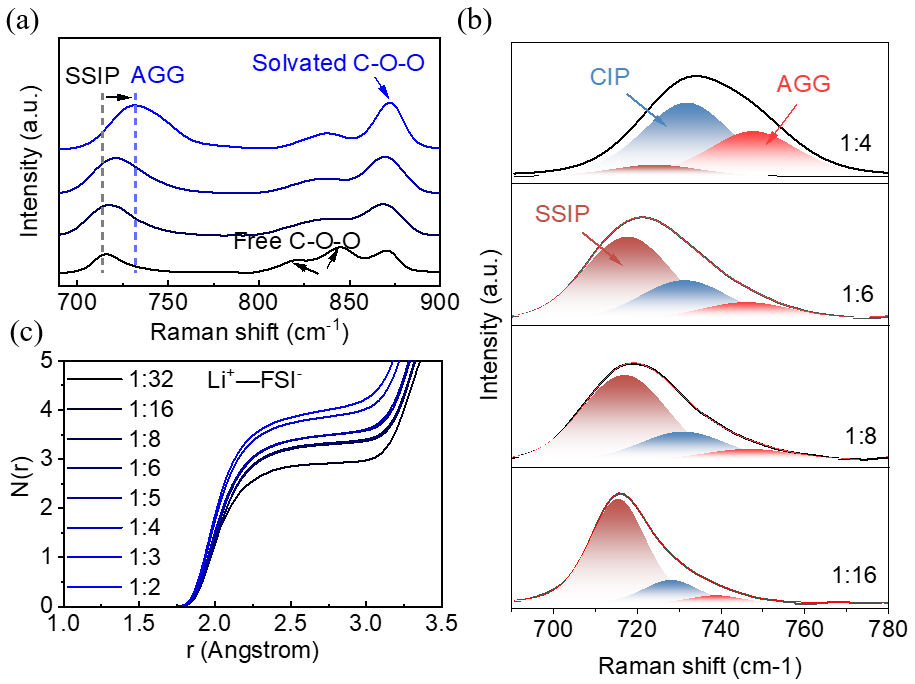


**Figure S29.** Raman analysis of LiFSI-DME electrolytes at varying salt concentrations. (a) Raman spectra in the 690–900 cm^-1^ range, illustrating solvation structure evolution with increasing LiFSI concentration (from bottom to top: Li: O = 1:16, 1:8, 1:6, 1:4). (b) Peak deconvolution of the Raman bands, identifying contributions from SSIP, CIP, and AGG species. (c) Radial distribution functions (RDFs) between Li^+^ and FSI^-^, confirming the trend of increasing anion coordination with concentration.


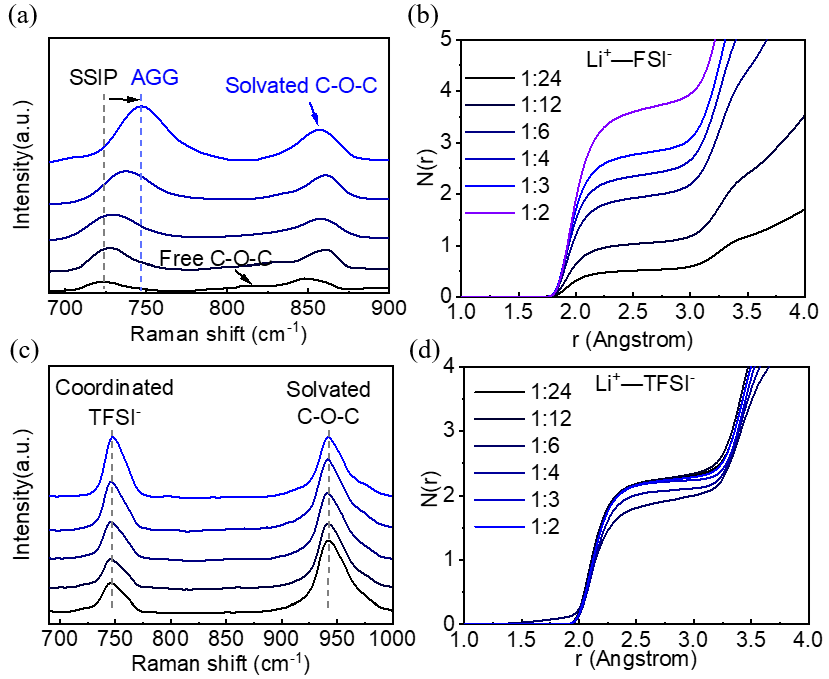


**Figure S30.** Raman spectroscopy and coordination number analysis of LiFSI-DEE and LiTFSI-DOL electrolytes. (a) Raman spectra of LiFSI–DEE in the 690–900 cm^-1^ range, showing peak shifts that reflect increasing Li^+^–FSI^-^ interactions with concentration. (b) Corresponding radial distribution functions (RDFs) between Li^+^ and FSI^-^, indicating a concentration-dependent increase in anion coordination. (c) Raman spectra of LiTFSI–DOL in the 690–1000 cm^-1^ range, displaying minimal spectral changes across concentrations. (d) RDFs for Li^+^–TFSI^-^ pairs, demonstrating relatively stable coordination behavior. In both (a) and (c), the sample order from bottom to top corresponds to Li: O ratios of 1:24, 1:12, 1:6, 1:4, 1:3, and 1:2.


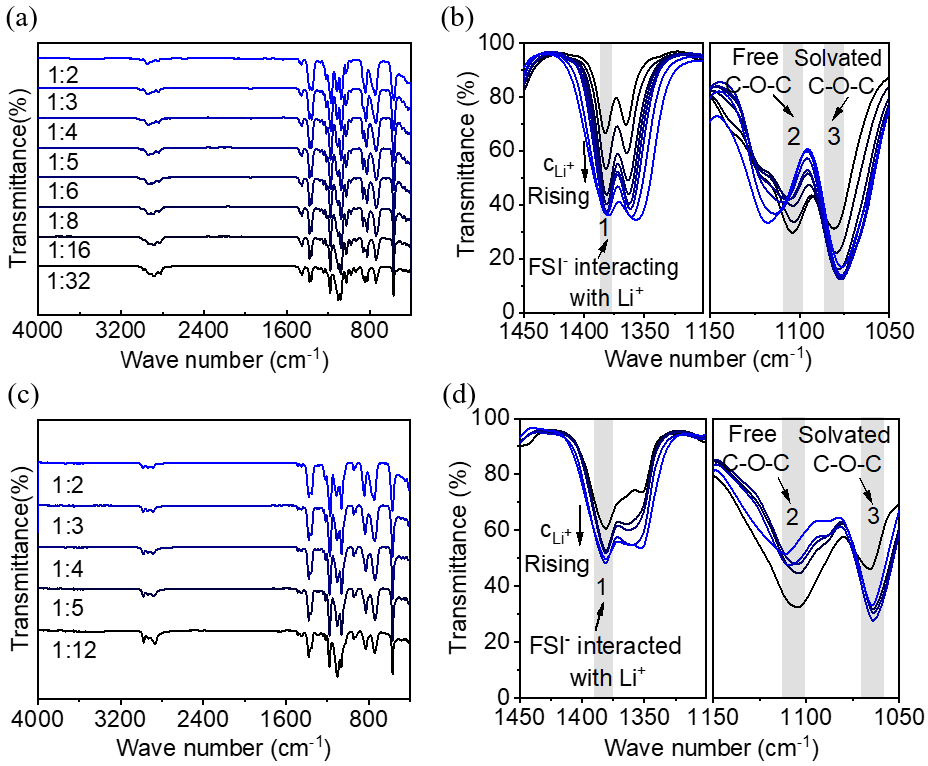


**Figure S31.** FTIR spectra of LiFSI-DME and LiFSI-DEE electrolytes at varying concentrations. (a, c) Full-range FTIR spectra of LiFSI-DME and LiFSI-DEE systems, respectively. (b, d) Enlarged views of the 1050–1150 cm^-1^ and 1300–1450 cm^-1^ regions, highlighting vibrational peaks attributed to Li^+^-coordinated FSI^-^, free FSI^-^, and Li^+^-coordinated solvent molecules. The observed spectral evolution with concentration supports the solvation structure trends predicted by MD simulations.

**Table S2.** Feature selection scores for the ACN dataset.

| Feature name | Pearson’ coefficient | RF  MIC | RF  R^2^ | Xgboost  Information Gain | GPR  ∆(MSE) | Sum of Scores | Final Selection |
| --- | --- | --- | --- | --- | --- | --- | --- |
|  |  |  |  |  |  |  |  |
| μ | √ | 2 | 4 | 10 | 1 | 17 |  |
| *E*_b_ | √ | 1 | 1 | 4 | 1 | 7 | √ |
| c_Li+_ | √ | 5 | 6 | 9 | 2 | 23 |  |
| sO | √ | 1 | 1 | 7 | 4 | 13 | √ |
| aC |  | 1 | 1 | 1 | 1 | 4 | √ |
| aO | √ | 1 | 1 | 3 | 2 | 8 | √ |
| aF | √ | 3 | 3 | 5 | 3 | 14 | √ |
| aN | √ | 6 | 7 | 6 | 2 | 21 |  |
| F/O |  | 1 | 1 | 2 | 2 | 6 | √ |
| sC |  | 4 | 5 | 11 | 3 | 23 |  |
| InOr | √ | 2 | 2 | 8 | 5 | 17 |  |
| Median |  | 3.5 | 4 | 6 | 3 | 16.5 |  |

**Table S3.** Feature selection scores for the SCN dataset.

| Feature name | Pearson’ coefficient | RF  MIC | RF  R^2^ | Xgboost  Information Gain | GPR  ∆(MSE) | Sum of Scores | Final Selection |
| --- | --- | --- | --- | --- | --- | --- | --- |
|  |  |  |  |  |  |  |  |
| μ | √ | 7 | 3 | 10 | 1 | 21 |  |
| *E*_b_ | √ | 1 | 1 | 2 | 1 | 5 | √ |
| c_Li+_ |  | 1 | 1 | 4 | 1 | 8 | √ |
| sO | √ | 1 | 1 | 3 | 3 | 8 | √ |
| aC | √ | 2 | 1 | 7 | 2 | 12 | √ |
| aO |  | 1 | 1 | 1 | 1 | 5 | √ |
| aF | √ | 8 | 4 | 11 | 3 | 26 |  |
| aN | √ | 6 | 5 | 9 | 3 | 23 |  |
| F/O | √ | 5 | 6 | 8 | 4 | 23 |  |
| sC | √ | 4 | 2 | 6 | 3 | 15 | √ |
| InOr |  | 3 | 1 | 5 | 4 | 14 | √ |
| Median |  | 4.5 | 3.5 | 6 | 2.5 | 16.5 |  |

**Table S4.** Feature selection scores for the *D*_Li_^+^ dataset.

| Feature name | Pearson’ coefficient | RF  MIC | RF  R^2^ | Xgboost  Information Gain | GPR  ∆(MSE) | Sum of Scores | Final Selection |
| --- | --- | --- | --- | --- | --- | --- | --- |
|  |  |  |  |  |  |  |  |
| μ | √ | 8 | 7 | 11 | 2 | 28 |  |
| *E*_b_ |  | 1 | 1 | 3 | 1 | 6 | √ |
| c_Li+_ | √ | 1 | 1 | 1 | 3 | 7 | √ |
| sO |  | 2 | 1 | 9 | 3 | 15 | √ |
| aC | √ | 4 | 3 | 10 | 2 | 19 |  |
| aO | √ | 1 | 1 | 2 | 2 | 7 | √ |
| aF | √ | 7 | 6 | 7 | 2 | 22 |  |
| aN | √ | 3 | 2 | 5 | 1 | 11 | √ |
| F/O | √ | 6 | 5 | 6 | 3 | 20 |  |
| sC |  | 5 | 4 | 8 | 4 | 21 |  |
| InOr | √ | 1 | 1 | 4 | 3 | 10 | √ |
| Median |  | 4.5 | 4 | 6 | 2.5 | 17 |  |

**Score standardization equation:**

V_stand_=$\left\{ \begin{aligned} \frac{V_{x}-V_{median}}{\left| {V_{min}-V}_{median} \right|}\times0.5+0.5, if V_{x}<V_{median} \\ \frac{V_{x}-V_{median}}{\left| {V_{max}-V}_{median} \right|}\times0.5+0.5, if V_{x}>V_{median} \end{aligned} \right.$ (S1)

Where V_stand_, V_min_, and V_median_ are the standardized, minimum, and median values of feature scores in each label column. Specially, when V_x_ = V_min_, V_stand_ is 0.01.


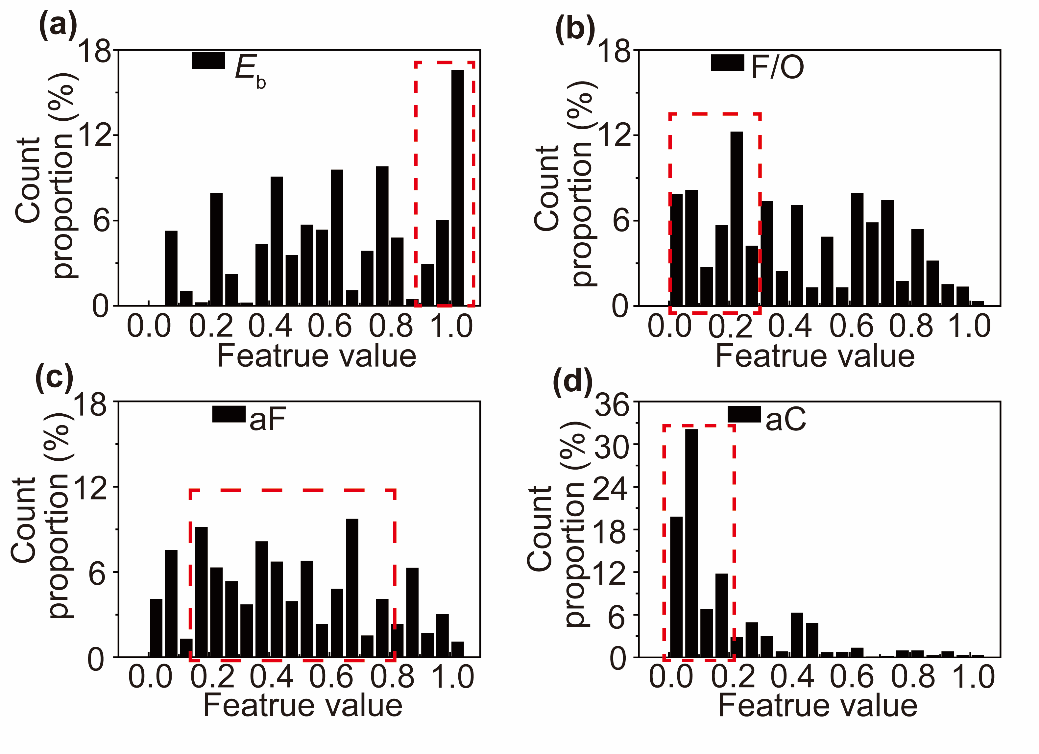


**Figure S32.** ACN intersected section data frequency count proportions. The frequency count proportions for feature values of *E*_b_ (a), F/O (b), aF (c), and aC (d) based on an intersected set of ACN, the section of concentrated distribution for the feature values were chosen as suggested section, specifically, *E*_b_: [0.9, 1], F/O: [0.05, 0.25], aF: [0.15, 0.75], aC: [0.05, 0.2].


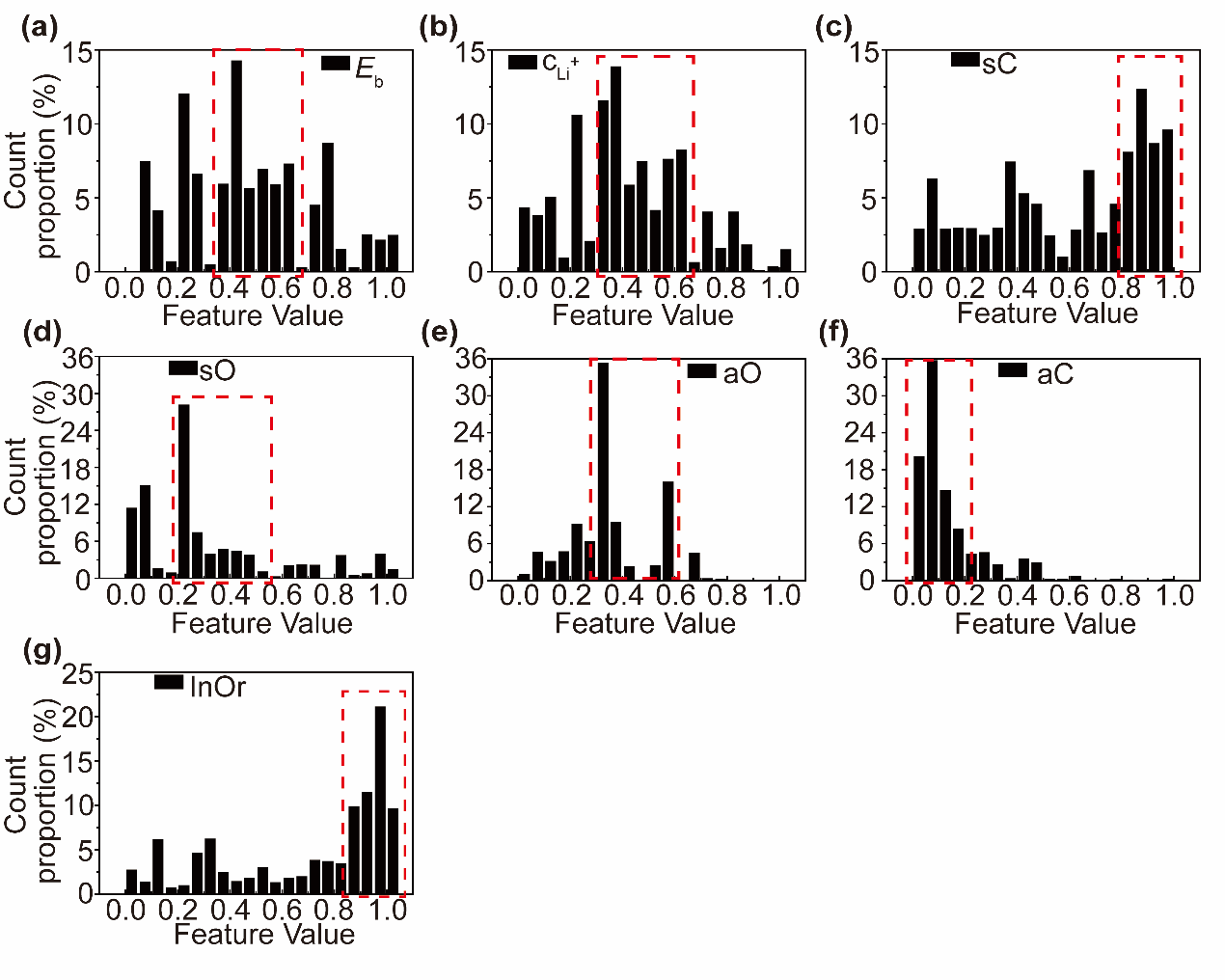


**Figure S33.** SCN intersected section data frequency count proportions. The frequency count proportions for feature values of *E*_b_ (a), c_Li_^+^ (b), sC (c), sO (d), aO (e), aC (f), and InOr (g) based on an intersected set of SCN, the section of concentrated distribution for the feature values was chosen as suggested section, specifically, *E*_b_: [0.35, 0.65], c_Li_^+^: [0.35, 0.65], sC: [0.85, 1], sO: [0.2, 0.55], aO: [0.25, 0.4], aC: [0.05, 0.2], InOr: [0.8, 1].


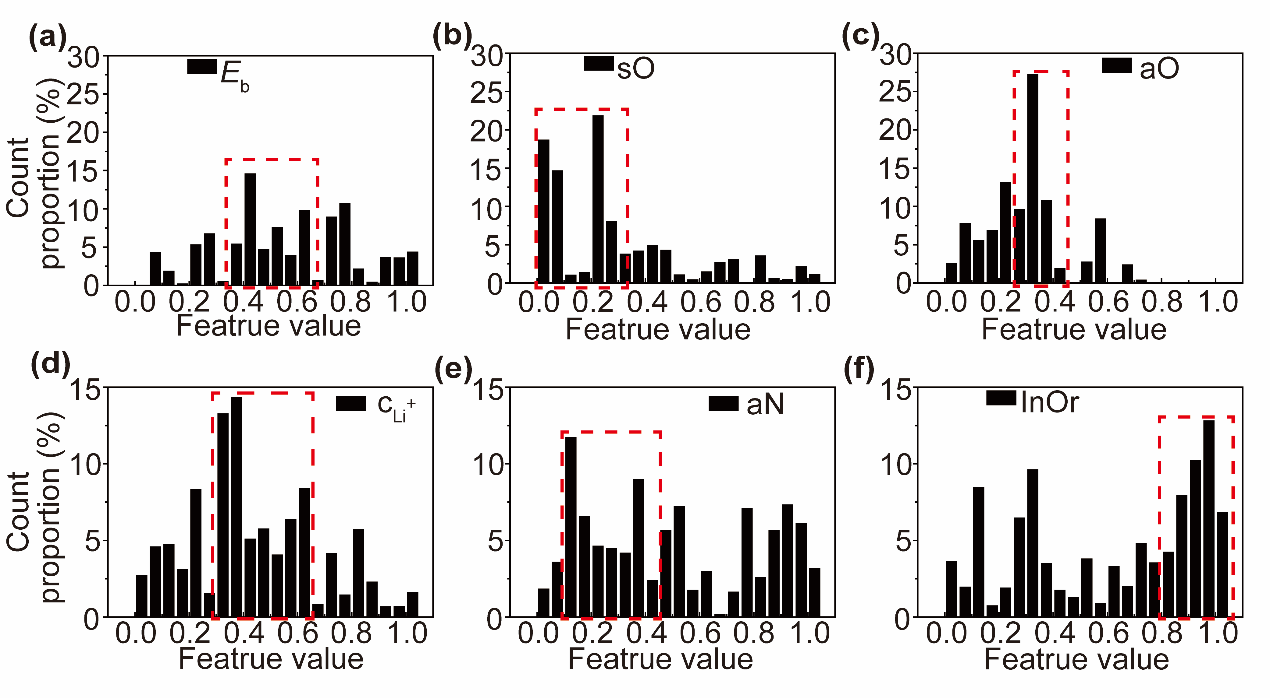


**Figure S34.** *D*_Li_^+^ intersected section data frequency count proportions. The frequency count proportion for feature values of *E*_b_ (a), sO (b), aO (c), c_Li_^+^ (d), aN (e) and InOr (f) based on intersected set of *D*_Li_^+^, the section of concentrated distribution for the feature values was chosen as suggested section, specifically, *E*_b_: [0.35, 0.65], sO: [0, 0.3], aO: [0.25, 0.4], c_Li_^+^ : [0.35, 0.65], aN: [0.1, 0.4], InOr: [0.8, 1].

**Table S5.** Electrolyte Scheme Design (g/5 ml).

|  | LiFSI | LiTFSI | LiDFOB | LiTFA | LiNFS | DEE | TREGDME | DIPE | Phenomena |
| --- | --- | --- | --- | --- | --- | --- | --- | --- | --- |
| S1 | 1.73 |  | 0.44 |  | 0.71 |  | 1.72 | 2.36 | Stratification |
| S2 | 1.73 |  |  | 1.20 |  |  | 1.72 | 2.36 | Stratification and freezing |
| S3 | 1.76 |  |  | 0.79 | 0.38 | 0.74 | 1.45 | 1.92 | Stratification |
| S4 | 2.28 |  |  | 0.33 | 0.97 | 2.50 | 0.71 | 0.95 | Freezing |
| S5 | 1.52 |  |  | 0.77 | 0.75 | 2.33 | 0.66 | 1.13 | Dissolved |
| S6 | 2.28 | 1.11 |  |  | 0.08 | 2.50 | 0.71 | 0.95 | Dissolved |
| S7 | 2.52 | 1.26 |  |  | 0.03 | 4.19 | 0.02 |  | Dissolved |
| S8 | 2.44 | 1.22 |  | 0.07 | 0.02 | 3.05 | 0.46 | 0.66 | Dissolved |
| S9 | 3.33 |  |  |  |  | 4.21 |  |  | Dissolved |
| S10 | 3.33 |  |  | 0.03 |  | 4.21 |  |  | Dissolved |
| S11 | 3.33 |  |  |  | 0.03 | 4.21 |  |  | Dissolved |

**Table S6.** Normalized vs. real conversion for feature values of the suggested section.

| Feature Name | lower bound (normalized) | lower bound (real) | upper bound (normalized) | upper bound (real) |
| --- | --- | --- | --- | --- |
| *E*_b_ | 0.4 | -101.661 (kcal/mol) | 0.6 | -80.84 (kcal/mol) |
| c_Li_^+^ | 0.35 | 0.191 (Li/O molar ratio) | 0.65 | 0.334 (Li/O molar ratio) |
| sO | 0.05 | 0.0323 | 0.3 | 0.0689 |
| aC | 0.05 | 0.00571 | 0.2 | 0.0229 |
| aO | 0.25 | 0.0503 | 0.4 | 0.0743 |
| aN | 0.1 | 0.00426 | 0.5 | 0.0213 |
| aF | 0.15 | 0.0446 | 0.75 | 0.195 |
| F/O | 0.05 | 0.156 | 0.25 | 0.597 |
| sC | 0.3 | 0.162 | 0.6 | 0.211 |
| InOr | 0.8 | 2.566 | 1 | 3.143 |


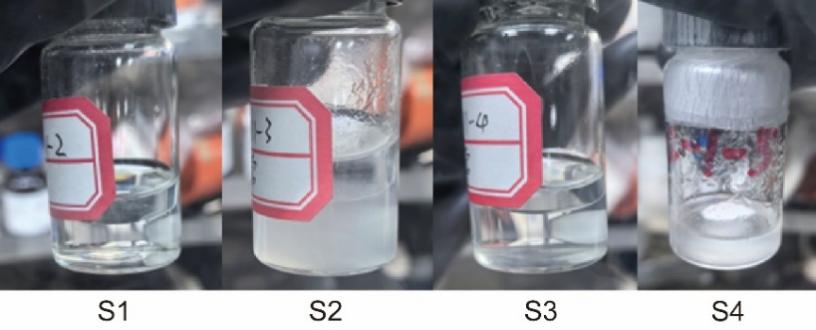


**Figure S35.** Freezing and stratification phenomena of 4 types of electrolytes. The stratification phenomena were observed in the S1 and S3 samples, and the freezing phenomenon was observed in the S4 sample, while the freezing and stratification phenomena were observed simultaneously in the S2 sample.


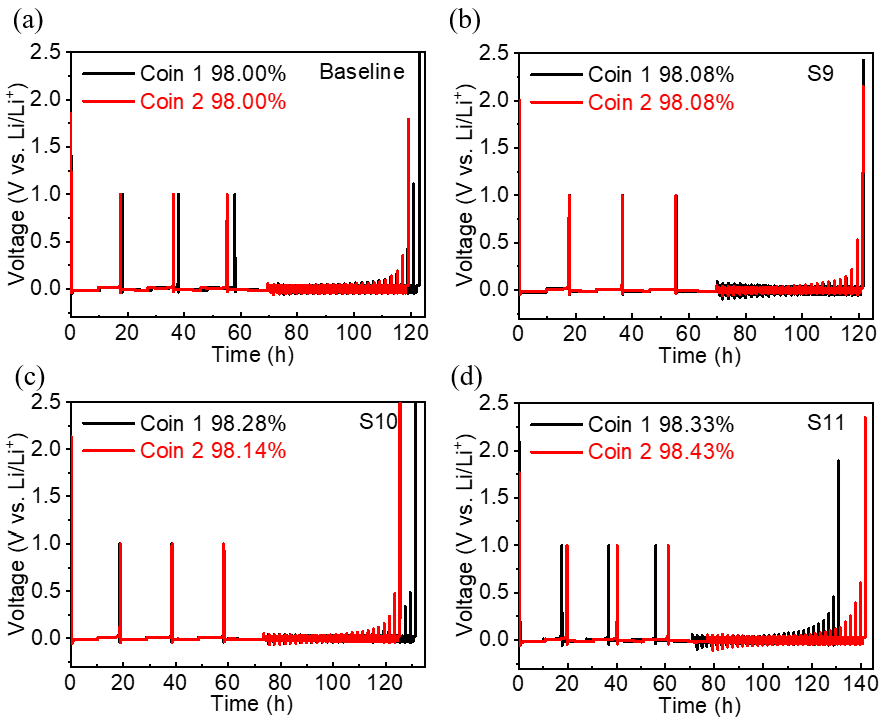


**Figure S36.** Evaluation of Coulombic efficiency (CE) using the Aurbach method for Li plating/stripping in Li‖Cu cells with different electrolytes. (a-d) Results for Baseline, S9, S10, and S11 electrolytes, respectively. Each test was performed with an initial plating capacity of 1 mAh cm^-2^ (0.1 C for 3 cycles), followed by Li deposition at 1.5 mAh cm^-2^ and stripping at 1 C to a cut-off voltage of 1.5 V. The measured CE values for Coin Cell 1 and Coin Cell 2 are indicated in each panel.


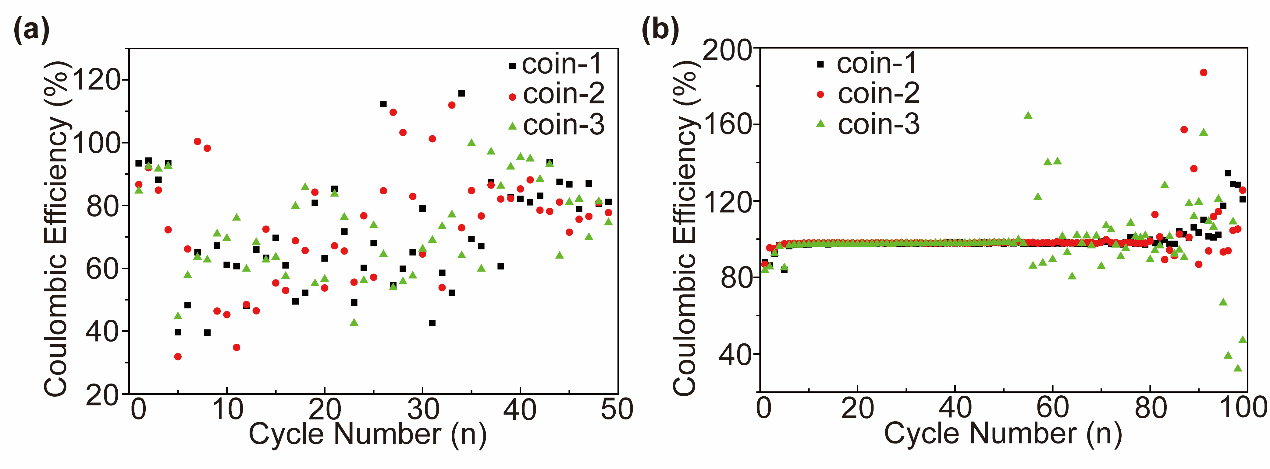


**Figure S37.** CE of S5(a) and S6(b). S5 appeared unstable CE in all 3 parallel samples, while S6 appeared rather low CE (~97.6%) and failed quickly around 80 cycles.


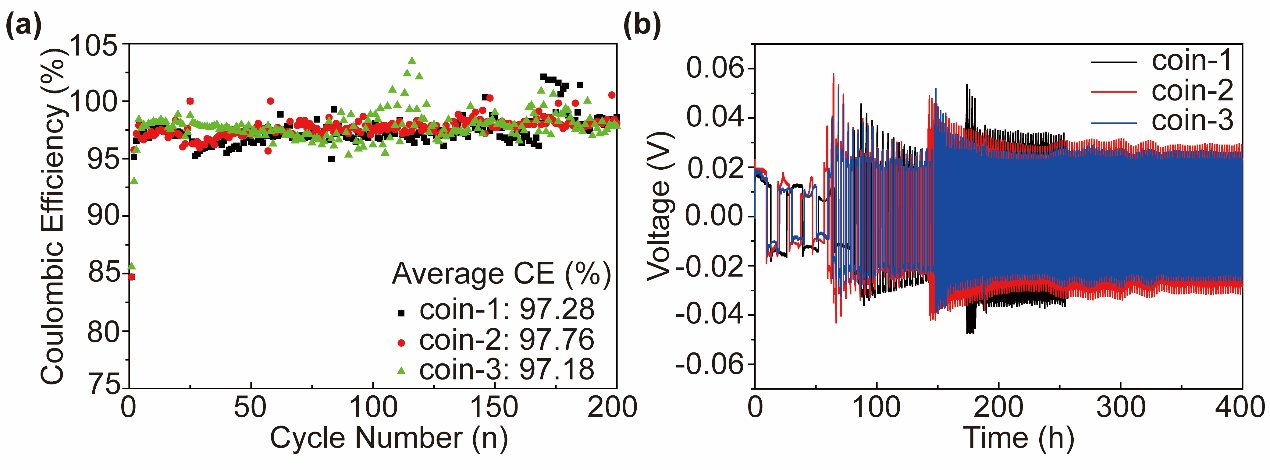


**Figure S38.** CE (a) and long-term cycling stability for lithium plating/striping (b) of S7. 3 parallel samples were measured for CE and long-term cycling, respectively.


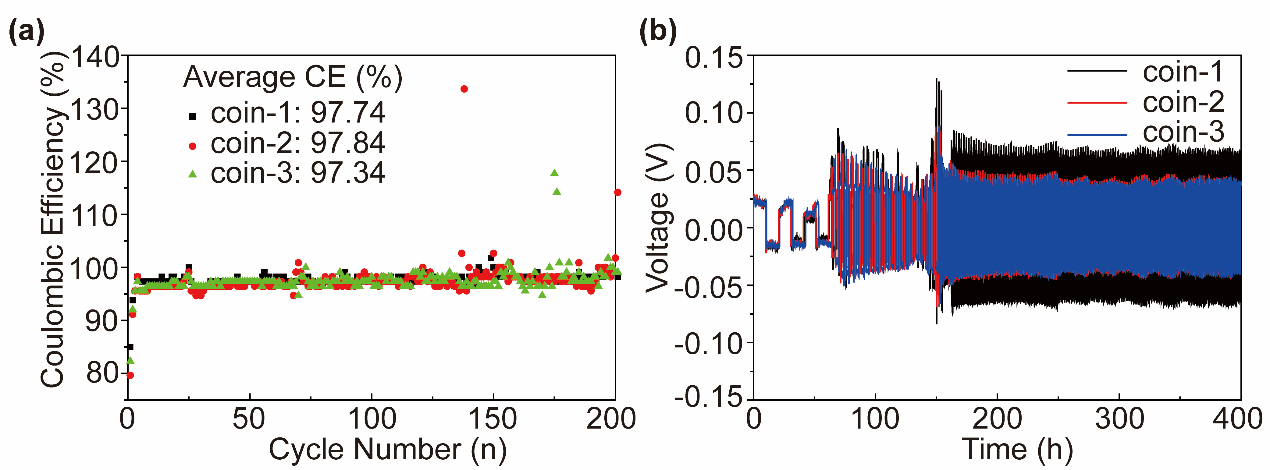


**Figure S39.** CE (a) and long-term cycling stability for lithium plating/striping (b) of S8.


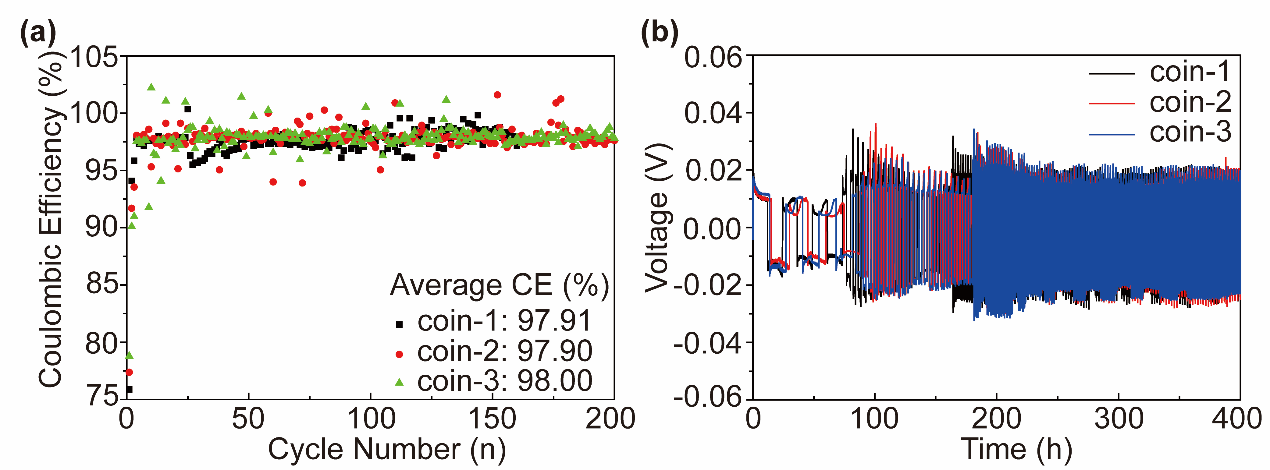


**Figure S40.** CE (a) and long-term cycling stability for lithium plating/striping (b) of S9.


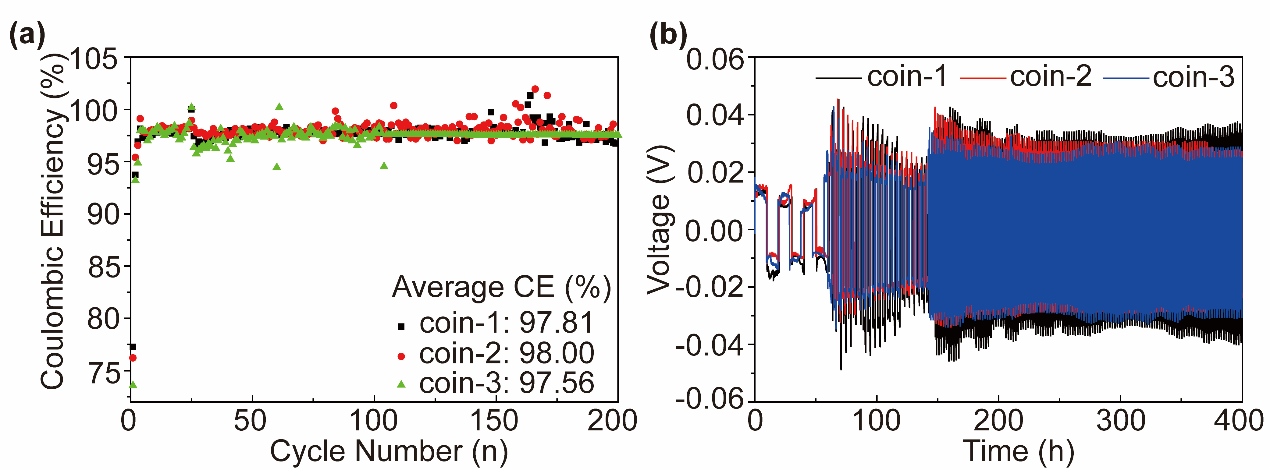


**Figure S41.** CE (a) and long-term cycling stability for lithium plating/striping (b) of S10.


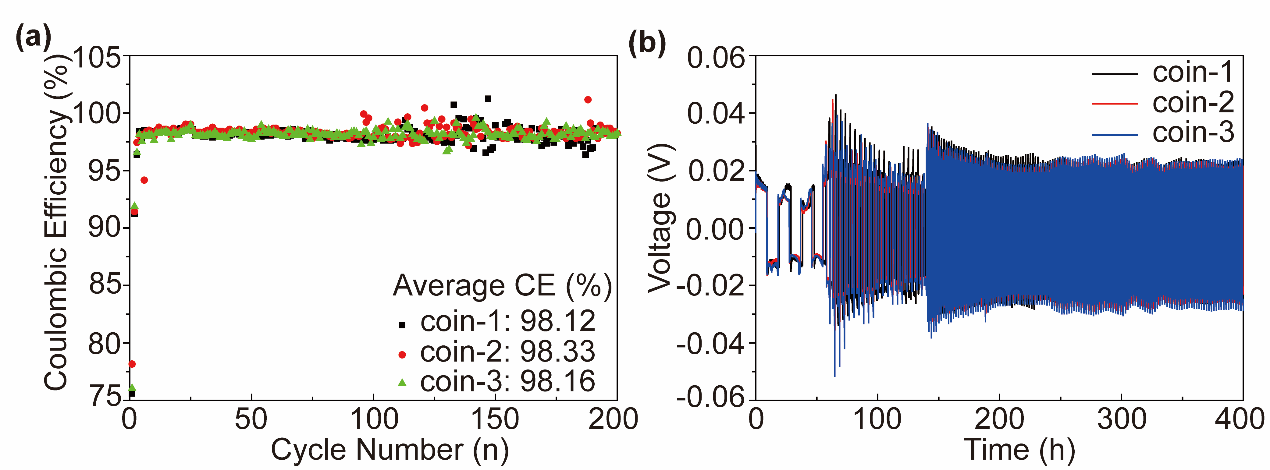


**Figure S42.** CE (a) and long-term cycling stability for lithium plating/striping (b) of S11.


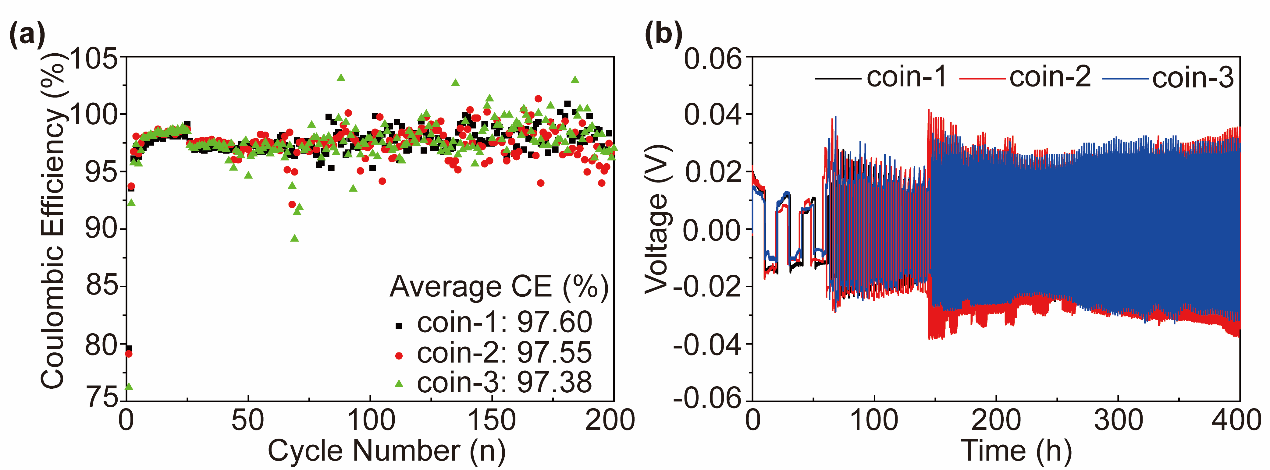


**Figure S43.** CE (a) and long-term cycling stability for lithium plating/striping (b) of the base sample.


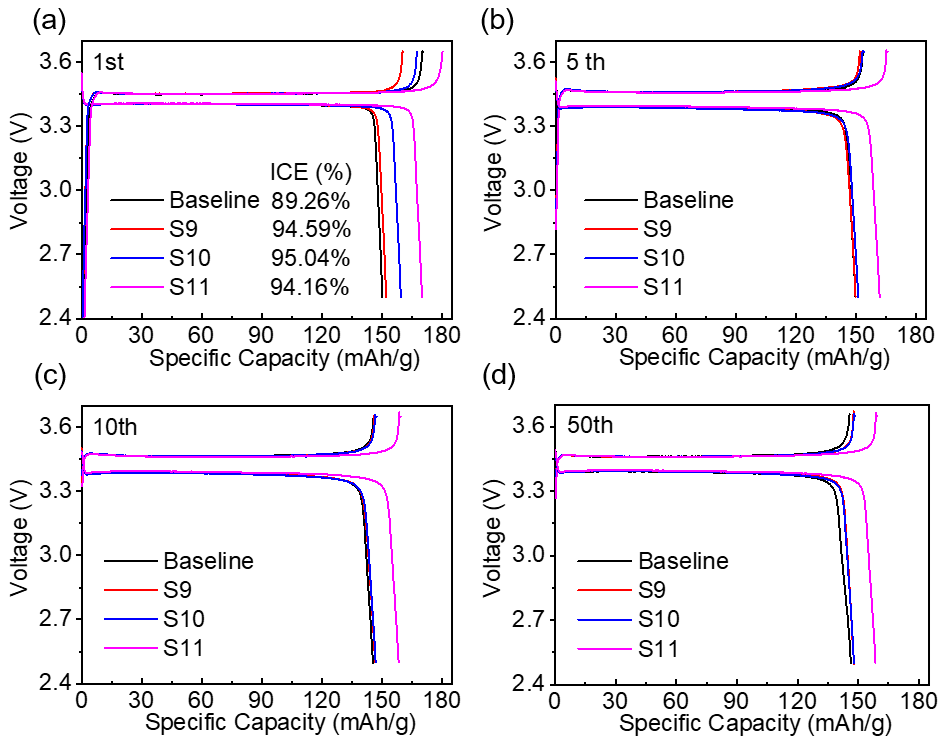


**Figure S44.** Voltage profiles at selected cycles for Baseline, S9, S10, and S11. (a-d) Voltage profiles at selected cycles for each system, showing improved initial Coulombic efficiencies and capacity retention in optimized electrolytes.

**Figure S45.** Feature values for base sample (“base” for short), and electrolytes S9, S10, and S11.

**Details for MD simulation**

System units were set as real, and the molecular forces were calculated using the Charmm (Chemistry at HARvard Macromolecular Mechanics) force field. The topology files, bonded, and Lennard-Jones parameters were generated using CGenFF Web App. The inner, outer, and coulombic interaction cutoff distances were set as 0.9, 1, and 1 nm, respectively. The particle-particle particle-mesh (PPPM) [1] method was used for long-range electrostatic interactions, and the accuracy was 10^-4^[2]. The skin distance was 0.35 nm with binning style. The neighbor lists were built for each step with 0 delay, and the timestep was set as 0.1 fs. To fully equilibrate the systems, the following procedures were performed: A 5 ps energy minimization using the conjugate gradient (CG) algorithm[3] method at 298 K, followed by a 5 ps production run using NVT ensemble at 298 K, then a 0.1 ns run using NPT ensemble at same temperature and pressure, after that the system was annealed for 1 ns using NPT ensemble, temperature was rising from 298 K to 363 K with 5 K per step. Then the system was equilibrated in the NPT ensemble at 298 K for another 0.2 ns. The following step was another anneal for 1 ns using the NVT ensemble, and the temperature rose from 298 K to 343 K with 5 K per step. The system was equilibrated in the NPT ensemble for another 0.2 ns at 298 K. The next step is equilibrating the run at 298 K in the NVT ensemble for 0.5 ns, followed by the last 0.5 ns run using the NVE ensemble. For all steps, the pressure was 100 kPa, and employing the Nosé-Hoover thermostat and barostat[4] for the temperature and pressure controlling method, the total simulation time is 3.5 ns. Periodic boundary conditions were applied in all directions. Density profiles and RDFs were generated using VMD[5].


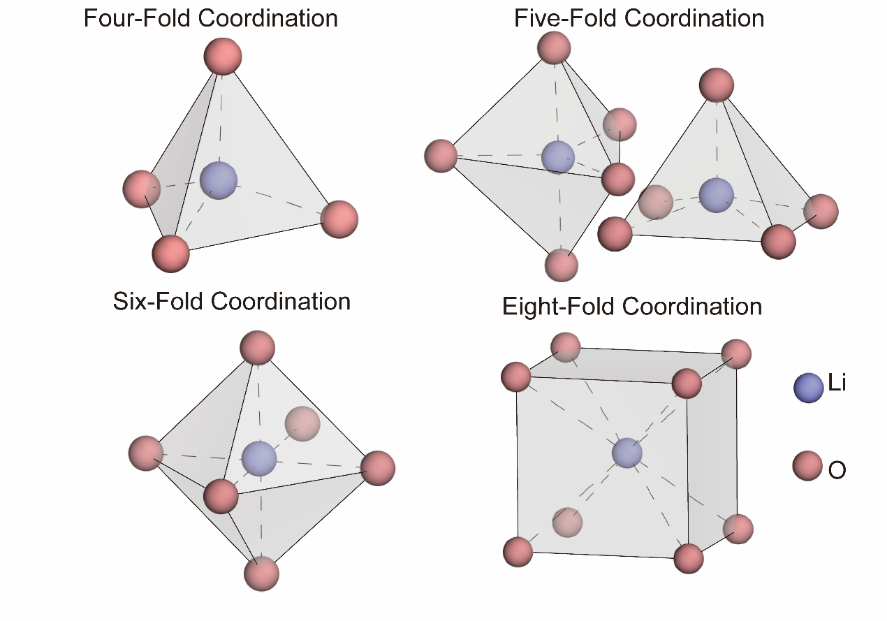


**Figure S46.** Coordination polyhedra.[6] The most probable coordinate circumstances for Li^+^ were shown, which were 4, 5, 6, and 8, respectively, where the five-fold coordination may exist in two situations. The atoms (all were supposed to be oxygen in this work) coordinated with Li^+^ were supplied by anion or solvent to form different clusters (AGG, CIP, and SSIP).


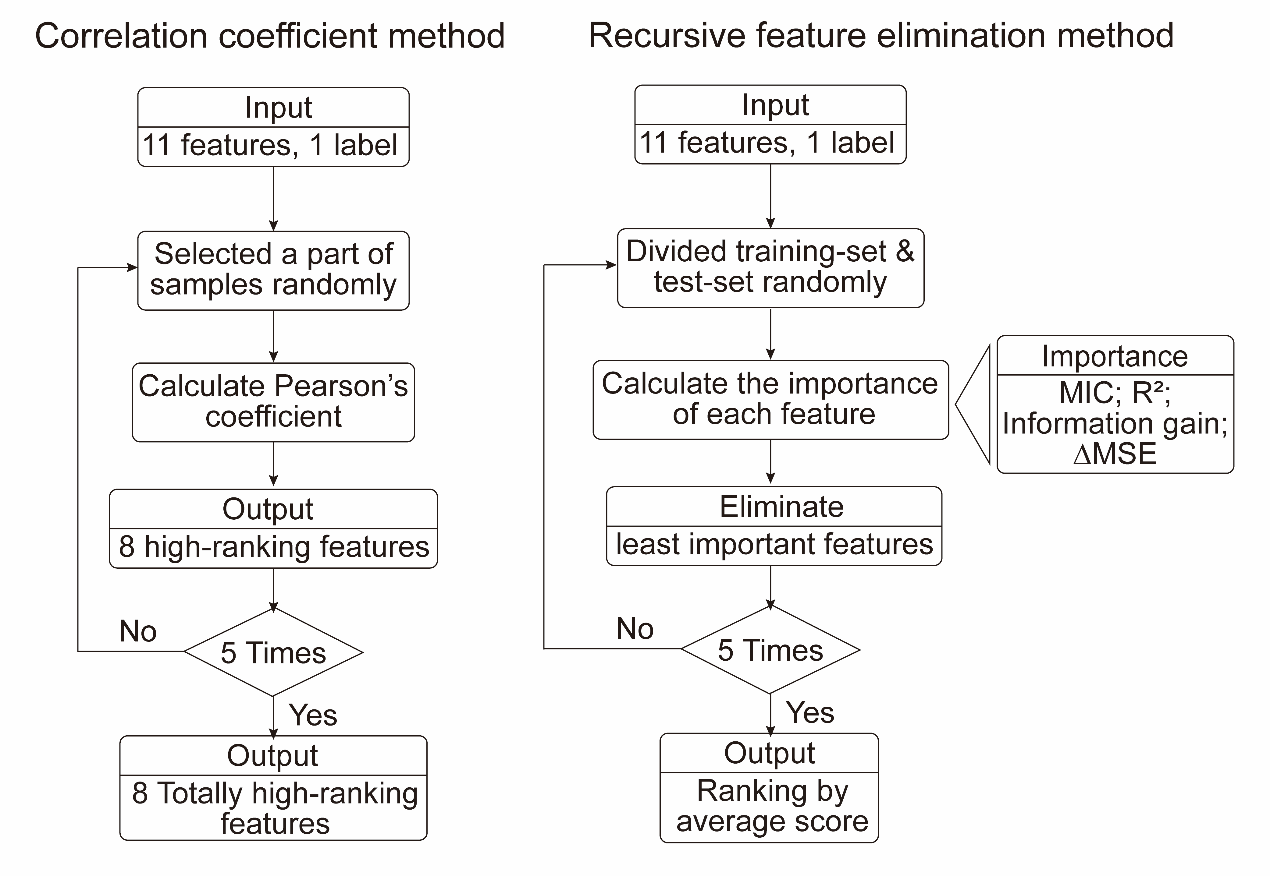


**Figure S47.** Feature selection flow chart of the two methods. The left one was the correlation coefficient method, 80% of samples were selected to calculate Pearson’s coefficient every loop, and the selection was performed 5 loops to choose the most related features; the right one was the recursive feature elimination method, the features were ranked according to their importance calculated by 4 kinds of criteria in the importance box.

Correlation coefficient (Pearson’s coefficient) equation:

$\rho_{X, Y}=\frac{cov(X, Y)}{\sigma_{X}\sigma_{Y}}=\frac{E[(X-EX)(Y-EY)]}{\sigma_{X}\sigma_{Y}}=\frac{E\left( XY \right)-E(X)E(Y)}{\sqrt{E\left( X^{2} \right)-E^{2}(X)} \sqrt{E\left( Y^{2} \right)-E^{2}(Y)}}$ (S2)

Mutual information coefficient (MIC) equation:

$I\left( X;Y \right)= \sum_{x, y} p\left( x, y \right)log\frac{p(x, y)}{p(x)p(y)}$ (S3)

Where p(x) and p(y) represent the probability of X=x_i_, and Y=y_i_, respectively. p(x, y) represents probability of X=x_i_ and Y=y_i_ appear simultaneously.

Determination coefficient(R^2^) equation:

$R^{2}=1- \frac{\sum_{i} {(\hat{y}_{i}-y_{i} )}^{2}}{\sum_{i} {(\bar{y}-y_{i})}^{2}}$ (S4)

The numerator represents the sum of the difference of squares between real values and predicted values; the denominator represents the sum of the difference between real values and mean values.

Information gain:

g(D, A) = H(D) - H(D|A) (S5)

where $H\left( D \right)= -\sum_{k=1}^{K} \frac{C_{k}}{D}{log}_{2}\frac{C_{k}}{D}$, $H_{A}\left( D \right)= - \sum_{i=1}^{n} \frac{\left| D_{i} \right|}{\left| D \right|}{log}_{2}\frac{\left| D_{i} \right|}{\left| D \right|}$

Information gain represents a reduction in the extent of indeterminacy of ŷ according to one feature, X, in the dataset.

∆MSE = | MSE_before_ – MSE_after_ | (S6)

∆MSE: The difference between MSEs calculated before and after eliminating one feature using the GPR model, the small variation means a small impact of the relevant feature on the model.

For Xgboost, the learning rate was chosen as 0.2, booster: gbtree, and max_leaves: 127; the RF parameter is trees: 500, max depth: 7, min_samples_split: 2, and min_samples_leaf: 1.

The GPR kernel function was built as equation 6.

kernel = *ConstantKernel*(constant_value=0.2, constant_value_bounds=(10^-4^, 10^4^)) * *RBF*(length_scale=0.4, length_scale_bounds=(10^-4^, 10^4^)) + *Matern*(length_scale=1.0, length_scale_bounds=(10^-3^, 10^3^), nu=1.5) (S7)


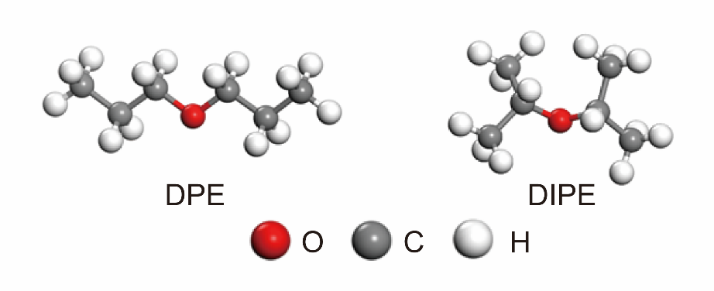


**Figure S48.** Comparison of the molecular structures of DPE and isomeric DIPE.

### Reference

[1] R. W. Hockney, J. W. Eastwood, *CRC Press* **1988**, <https://doi.org/10.1201/9780367806934>.

[2] Z. Li, H. Rao, R. Atwi, B. M. Sivakumar, B. Gwalani, S. Gray, K. S. Han, T. A. Everett, T. A. Ajantiwalay, V. Murugesan, N. N. Rajput, V. G. Pol, *Nat Commun* **2023**, *14* (1), 868, <https://doi.org/10.1038/s41467-023-36647-1>.

[3] M. R. Hestenes, E. Stiefel, *Journal of Research of the National Bureau of Standards* **1952**, *49* (6), <https://doi.org/10.6028/JRES.049.044>.

[4] a) S. Nosé, *J Chem Phys* **1984**, *81* (1), 511~519, <https://doi.org/10.1063/1.447334>; b) S. Melchionna, G. Ciccotti, B. Lee Holian, *Mol Phys* **2006**, *78* (3), 533~544, <https://doi.org/10.1080/00268979300100371>.

[5] <https://doi.org/> <https://www.ks.uiuc.edu/Research/vmd/>.

[6] U. Olsher, R. M. Izatt, J. S. Bradshaw, N. K. Dalley, *Chem Rev* **2002**, *91* (2), 137~164, <https://doi.org/10.1021/cr00002a003>.

1. a These authors contributed equally to this work. [↑](#footnote-ref-0)
2. * Corresponding authors, E-mail: liaijun@gfenergy.com; yfzhu@zstu.edu.cn [↑](#footnote-ref-1)
